# Spatial and temporal patterns of nitric oxide diffusion and degradation drive emergent cerebrovascular dynamics

**DOI:** 10.1371/journal.pcbi.1008069

**Published:** 2020-07-27

**Authors:** William Davis Haselden, Ravi Teja Kedarasetti, Patrick J. Drew

**Affiliations:** 1 Neuroscience Graduate Program, MD/PhD Medical Scientist Training Program, Pennsylvania State University, University Park, Pennsylvania, United States of America; 2 Department of Engineering Science and Mechanics, Pennsylvania State University, University Park, Pennsylvania, United States of America; 3 Departments of Biomedical Engineering and Neurosurgery, Pennsylvania State University, University Park, Pennsylvania, United States of America; Stanford University, UNITED STATES

## Abstract

Nitric oxide (NO) is a gaseous signaling molecule that plays an important role in neurovascular coupling. NO produced by neurons diffuses into the smooth muscle surrounding cerebral arterioles, driving vasodilation. However, the rate of NO degradation in hemoglobin is orders of magnitude higher than in brain tissue, though how this might impact NO signaling dynamics is not completely understood. We used simulations to investigate how the spatial and temporal patterns of NO generation and degradation impacted dilation of a penetrating arteriole in cortex. We found that the spatial location of NO production and the size of the vessel both played an important role in determining its responsiveness to NO. The much higher rate of NO degradation and scavenging of NO in the blood relative to the tissue drove emergent vascular dynamics. Large vasodilation events could be followed by post-stimulus constrictions driven by the increased degradation of NO by the blood, and vasomotion-like 0.1–0.3 Hz oscillations could also be generated. We found that these dynamics could be enhanced by elevation of free hemoglobin in the plasma, which occurs in diseases such as malaria and sickle cell anemia, or following blood transfusions. Finally, we show that changes in blood flow during hypoxia or hyperoxia could be explained by altered NO degradation in the parenchyma. Our simulations suggest that many common vascular dynamics may be emergent phenomena generated by NO degradation by the blood or parenchyma.

## Author summary

Nitric oxide (NO) generated by neurons during states of increased neural activity dilates arteries, increasing local cerebral blood flow. Scavenging of nitric oxide is much more rapid in the blood than in the tissue, but how the dynamics of NO production and degradation affect the coupling between neural activity and vasodilation is not understood. Here we used computer simulations to model how nitric oxide produced by neurons leads to changes in arteriole size. We find that because nitric oxide is removed from the brain by the blood, changes in arteriole size or blood composition play a role in shaping neurally evoked changes in cerebral blood flow. As the arteriole dilates and supplies more blood, nitric oxide is removed by the blood at a faster rate and this interaction is able to reproduce many commonly observed arteriole dynamics. These dynamics can also be affected by pathologies where blood cells break down, providing a potential link between the state of the blood and blood flow dynamics in the brain.

## Introduction

Increases in neural activity in the brain typically are followed by the dilation of nearby arterioles [1–6] and potentially capillaries [7,8], (although this is still controversial [9]). The dilation of these vessels lowers the local vascular resistance, leading to a local increase in blood flow and oxygenation that is the basis of many brain imaging techniques [6,10,11]. The relationship between neural activity and these hemodynamic signals is known as neurovascular coupling (NVC). The maintenance of adequate coupling is thought to play a critical role in brain health [12]. In some cases, the vasodilation driven by increased neural activity is followed by a post-stimulus arterial constriction below baseline that results in a reduction of blood volume and blood flow below the pre-stimulus baseline. This is known as the post-stimulus undershoot [13–15]. This post-stimulus undershoot is not always observed, and its origin is not understood [16]. In addition to the post stimulus undershoot, arteries show spontaneous oscillations in diameter in the 0.1–0.3 Hz range, known as vasomotion [17–23], whose origin is not understood. Thus, in addition to dilations linked to increases in neural activity, cerebral arterioles show a wide range of dynamic behaviors.

Multiple signaling pathways have been implicated in coupling neural activity to increases in blood flow [24]. Signals from astrocytes [8,25–27] and neurons [28–33] are both thought to contribute to driving neurovascular coupling. One pathway implicated in neurovascular coupling is nitric oxide (NO) [34–36]. NO is vasoactive [37] and affects neural excitability as well [38]. NO diffuses through aqueous and lipid mediums [39,40], allowing for temporally and spatially complex signaling dynamics [41–43]. NO is produced by three types of nitric oxide synthases (NOS) [44,45]. The neuronal NOS (nNOS or NOS1) subtype of NOS is expressed by neurons [46], while endothelial cells express endothelial NOS [47] (eNOS or NOS3), and synthesis of NO by both enzymes is coupled to intracellular calcium [48]. An inducible, non-calcium dependent form of NOS is found in macrophages and other cells [49] (iNOS or NOS2), and is not found in the healthy brain. NO activates guanylyl cyclase (GC) in nearby cells to produce a rise in cGMP [50] and elicit vasodilation [39,51–54]. Despite the importance of NO in neurovascular coupling, *in vivo* measurements of NO levels in the brain have remained technically challenging. The recruitment of iNOS during injury and the non-specificity of probes [55] may account for the large range in NO concentration reported in the literature [56]. At high concentrations, NO will block respiration in mitochondrial cytochrome c oxidase (CcO), and result in cellular damage from inhibited respiration and free radical formation [57,58]. Because of this toxic effect on mitochondrial respiration, there will be an upper bound on NO levels in the healthy brain. Understanding the role of NO in neurovascular coupling is a topic of ongoing research [8,12,35,56,59–68]. Increases in NO production precede functional hyperemia [61], and modulation of NO availability alters baseline vessel diameter [36,63]. Inhibition of NO production blunts or abolishes the hemodynamic response [34,35,69] and causes reduction in baseline blood flow [36]. Finally, optogenetic stimulation of neuronal nitric oxide synthase positive interneurons, but not other interneurons, causes vasodilation without detectable changes in the activity of other neurons [70,71]. NO has been speculated to play a modulatory rather than a direct role in neurovascular coupling because functional hyperemia is attenuated by NOS inhibition and rescued by application of a NO donor, suggesting that the presence of NO allows functional hyperemia to occur [63]. However, NO has a role in increasing neuronal excitability [72–76], making the interpretation of these results difficult.

NO levels will depend not only on the dynamics of NO production, but also the degradation rate. In the tissue, NO degradation is proportional to the partial pressure of oxygen, so levels of NO will tend to inversely vary with tissue oxygenation [77,78]. However, the majority of NO is scavenged by hemoglobin in the blood which can do so a thousand-fold faster than the surrounding tissue [77,79–82]. Because NO reacts with hemoglobin at much higher rates than the tissue, the hemoglobin present inside a vessel plays an appreciable role in shaping NO concentrations at the smooth muscle where it acts. Under normal conditions, most hemoglobin in the blood in confined to red blood cells, with low levels in the plasma. Due to hydrodynamic interactions between red blood cells and the walls of the blood vessel [83–86], red blood cells will be excluded from the few micrometer-thick cell free layer next to the endothelial cells, providing a measure of spatial separation between the region of high NO degradation and the smooth muscle. However, if hemoglobin levels in the plasma rise (due to pathology or aging) [87–94], this will greatly increase the degradation rate of NO in the plasma, leading to decreased NO levels in the smooth muscle [88,95–97]. NO’s diffusive properties and known reaction rates lend themselves to computational approaches to understanding NO signaling [39,60,79,82,98–103]. While there have been detailed and informative models of NO signaling from endothelial cells [60,96,101,104,105] showing that the size of the arteriole [79] and properties of the blood [101] are vital components to understanding NO signaling, the insight from these models that the size of the arteriole plays an important role in the degradation of NO has not been applied to neurovascular coupling in a dynamic setting.

Intriguingly, *in vitro* experiments have shown that NO released by endothelial cells can depolarize axons [68], and flow changes in vessels can alter interneuron activity [66], potentially providing a mechanism by which vascular cells can modulate neural activity. The idea of bidirectional signaling between neurons and the vasculature (‘hemo-neural’ hypothesis [106,107]) has been put forward, though there is no definitive candidate mechanism. Signaling through NO-dependent pathways is a possible mechanism for hemo-neural coupling, as NO is known to affect neural excitability, and the amount of blood present could impact NO levels in the parenchyma through scavenging.

In order to understand how neuronal sources of NO communicate with the vasculature, we simulated NO production around a penetrating arteriole. In this model, the diameter of the vessel was dynamically dilated in response to the levels of NO present in the smooth muscle. We found that the sources of NO needed to be close to the arteriole to prevent inhibition of mitochondrial respiration. The increased amount of hemoglobin present during dilation greatly increased the removal of NO, which drove arteriole dynamics such as vasomotion and a post-stimulus undershoot. The concentration of plasma free hemoglobin in the blood was able to alter these vasodynamics. NO was able to function as an oxygen sensor in our model because its rate of removal in the parenchyma is dependent on the partial pressure of oxygen in the tissue. Finally, simulations imposing increases in vessel diameter when NO production rates were not varied resulted in a decrease in NO levels in the parenchyma, suggesting a potential mechanism for hemo-neural coupling. These results suggest that the diffusion and degradation of NO can drive emergent vascular dynamics.

## Results

### Model summary

**Fig 1:**
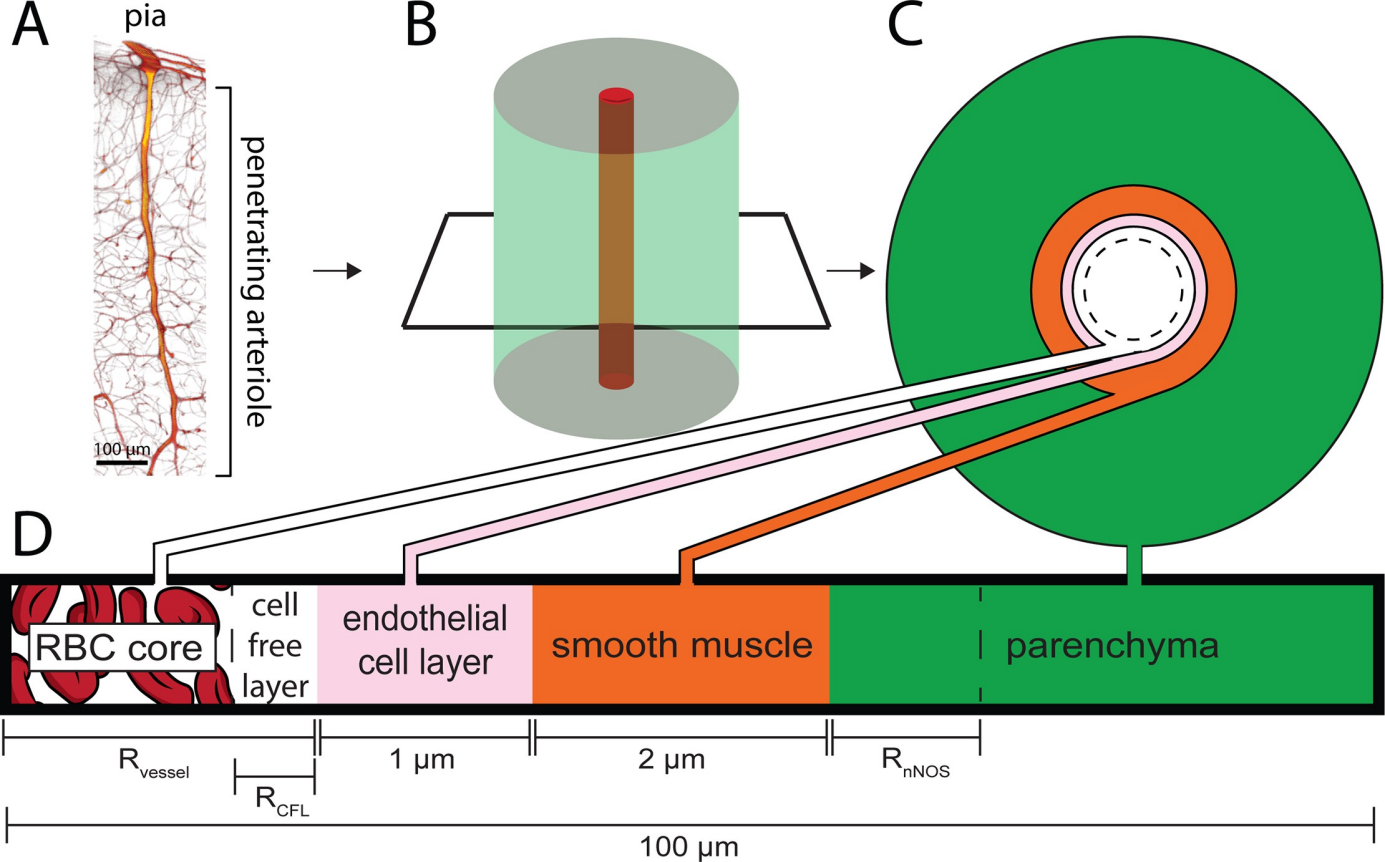
Schematic of the model. A) 3D reconstruction from serial 2-photon tomography of a penetrating arteriole. Penetrating arterioles are oriented perpendicularly to the pial surface. B) Simplified geometry used in the simulation where the penetrating arteriole is modeled as single arteriole surrounded by a cylinder of parenchymal tissue. C & D) Locations and thicknesses of the domains in the model. At the center are red blood cells (RBC core). The cell free layer is a thin layer of plasma lacking red blood cells immediately adjacent the endothelial cell layer. Both the RBC core and the cell-free layer size are dynamically changed when the vessel dilates or constricts. The endothelial cells and smooth muscle make up the arterial wall, and the vessel radius is taken to be the distance from the center of the vessel to the inner wall of the endothelial cells. Outside the smooth muscle is the parenchyma, composed of neurons, glia and extracellular space. The simulated tissue cylinder is 100 μm in diameter. The thickness of the NO-synthesizing portion of the tissue (R_nNOS_), vessel diameter (R_vessel_) and the size of the cell free layer (R_CFL_) were parametrically varied.

**Fig 2:**
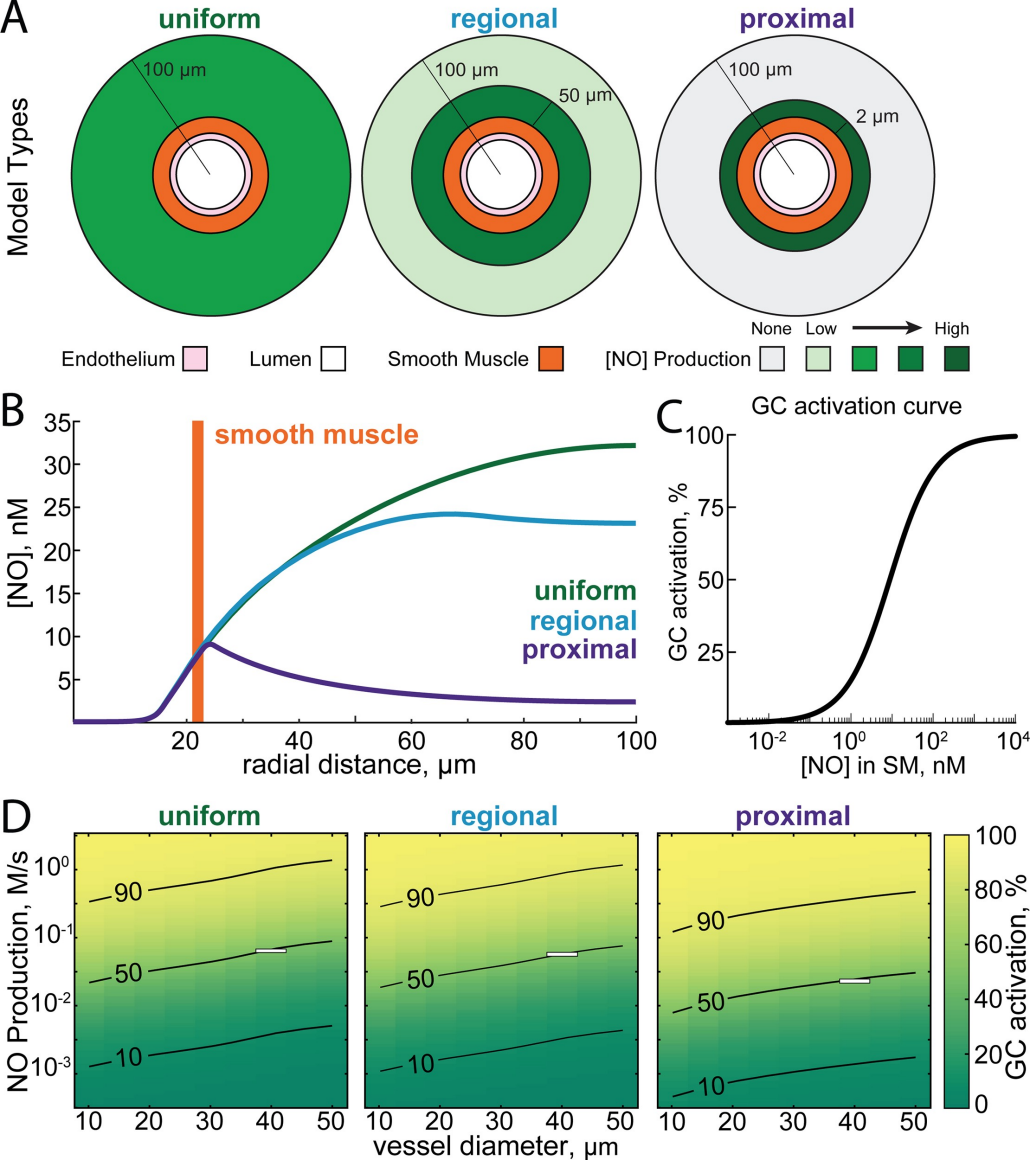
Impact of the location of NO production on NO concentration in the smooth muscle and tissue. All simulations in this figure use a static model. A) Schematic showing the three simulated distributions of neuronal NO production relative to the vasculature. In the uniform model, neuronal NO-production is uniformly distributed through the parenchyma. In the regional model, there is a higher density of neuronal NO production near the vessel (within 50 μm) [33]. In the proximal model, all neuronal NO is produced within 2 micrometers of the arterial wall [33,122,123]. B) Plot of NO concentrations versus radial distance for each of the three models where the production rates have been chosen to yield equal concentration of NO in the smooth muscle layer (NO production rate for proximal: 0.02 M/s; regional: 0.05 M/s; uniform: 0.056 M/s). Note that the concentration of NO in the parenchyma is very different for each of these models. C) Relationship between [NO] in the smooth muscle and percent of maximal guanylyl cyclase activity in the model, based on experimental data in [51,117,118]. D) Plot showing percent of maximal guanylyl cyclase activation in the smooth muscle as a function of the NO production rate and vessel diameter in each of the three geometries. Superimposed curves show 10, 50, and 90% of maximal guanylyl cyclase activation. White boxes show the NO production rates and vessel diameters shown in B.

**Fig 3:**
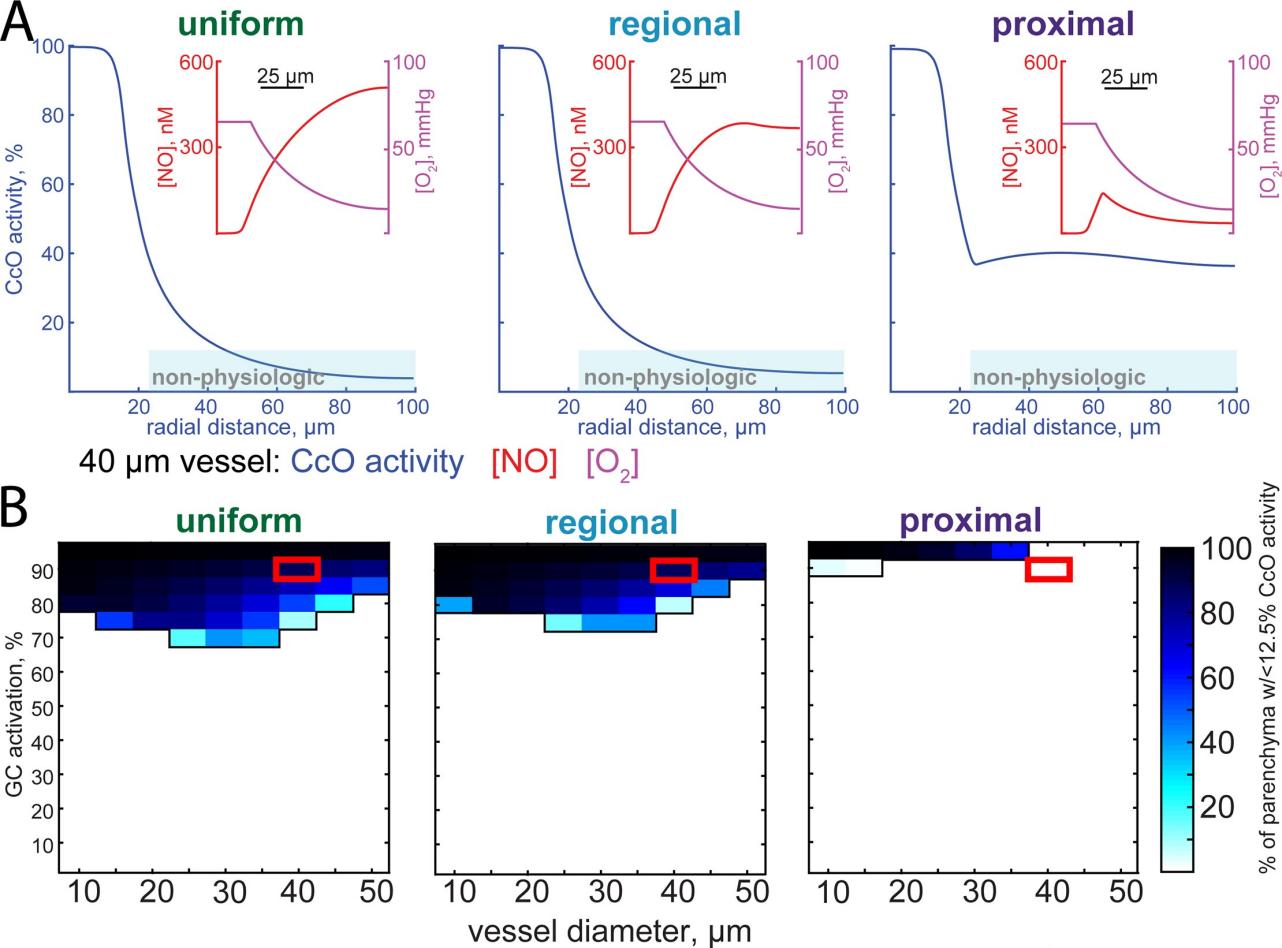
Extent of the NO inhibition of mitochondrial respiration depends on the location of NO production. All simulations in this figure use a static model. A) Plots of cytochrome c oxidase (CcO) activity as a function of radial distance for the uniform, regional, and proximal models. The vessel diameter was fixed at 40 micrometers, and NO production rates have been set so that there is 90% of maximal GC activation in the smooth muscle. Insets show oxygen and NO concentrations as a function of radial distance. Note that NO levels in the proximal model are not monotonically increasing with distance but show a peak around 25μm (see inset), which accounts for the shape of the CcO inhibition curve in the proximal model. Oxygen concentration curves are set to match *in vivo* measurements [130,131]. B) The fraction of the parenchyma (excluding the lumen, endothelial, and smooth muscle) where CcO activity is inhibited to <12.5% of normal as function of various NO production levels and vessel diameters for each of the three different NO production geometries. Red boxes indicate simulations plotted in (A). Note that for the regional and uniform NO production geometries, CcO inhibition becomes an issue at a wider range of NO production levels. For the proximal production case, pronounced inhibition of respiration by NO only occurs at the highest levels of NO production.

The model used here is of a penetrating arteriole in the cortex. In the cortex, penetrating arteries enter into the parenchyma perpendicular to the pial surface, and supply blood to a cylindrical volume of brain tissue approximately a hundred microns in radius [108–112] (**Fig 1A**). Because the geometry of the vasculature is complex and variable [113,114] and most of the branches off the arteriole are found in the deeper layers of cortex, we simplified the geometry to a single penetrating arteriole surrounded by a cylinder of neural tissue (**Fig 1B**). We simulated the production of and degradation of NO in a cylinder of tissue 100 μm in radius and 400 μm in length. The diameter of the penetrating arteriole is an important (but not the only) regulator of local blood flow [8,115,116]. In the model, the arteriole is surrounded by a domain of smooth muscle and the concentration of NO in the smooth muscle contributes to arteriole diameter. The model consisted of five domains, the red blood cell (RBC) core, the cell free layer, the endothelial cells, smooth muscle and the parenchyma (**Fig 1C and 1D**). Each domain has an associated NO production and/or degradation rate, and NO is produced in both the endothelial cell domain and the parenchyma. The thicknesses of the RBC core and cell-free domain were specified for each diameter according to empirical fits from published data [83,84]. Unless otherwise specified, the concentrations of NO and oxygen were calculated using Fick’s equation and the Krogh model, respectively. The parenchyma was treated as a nearly incompressible linear elastic solid, and the NO production was normalized such that the small dilation-induced compression of the parenchyma did not introduce changes in parenchymal NO production rates (see Methods). For the simulations in **S1–S3 Figs**, we include convection of NO by the blood, in all other simulations we do not include convection. For the simulations shown in **Figs 2 and 3** and **S4 and S5 Figs**, the diameter of the artery was static, meaning that changes in NO concentration in the smooth muscle did not cause changes in vessel diameter. In all other simulations, the arterial diameter is dynamic, so changes in NO concentration in the smooth muscle cause changes in vessel diameter, which will produce corresponding changes in the amount of hemoglobin present. The dynamics of the change in diameter is controlled by the hemodynamic response function (HRF), which is convolved with the concentration of the arterial smooth muscle to give the change in vessel diameter. The amplitude of any diameter changes in response to smooth muscle NO changes is controlled by the scaling parameter ‘m’. We used an EC_50_ for GC activation of 8.9nM, as several studies [51,117–119] have yielded EC_50_ values in this range. We assumed that there is a baseline level of GC activation of 50% in vivo because there is ongoing, spontaneous neural activity in vivo [120] in the absence of a stimulation which will produce a baseline level of NO. Inhibition of nNOS activity in vivo causes decreases in flow [121], indicating that there a tonic level of NO activity. **Table 1**shows all the parameters of the model, their physical meaning, and cites the literature from which they were taken.

**Table 1:** Simulation Parameters.

Geometry Variable	Value	Ref
***R*_*vessel*_ (vessel radius)**	5–25 μm	
***R*_*CFL*_ (thickness of cell-free layer)**	1.5–4.3 μm	[83,84]
***R*_*nNOS*_ (thickness of NO producing region)**	2 μm (proximal) 50 μm (regional) N/A (uniform)	[33,122,123] [33]
**Domain production/degradation equations**	*R*	
**RBC Core**	−*k*_*RBC Hb*_*Hct*[*NO*][*Hb*_*RBC*_]−*k*_*CFL Hb*_(1−*Hct*)[*NO*][*Hb*_*CFL*_]	
**Cell Free Layer**	$-k_{{Hb}_{CFL}}[NO][{Hb}_{CFL}]$	
**Endothelial Cell Layer**	55∙10^−3^*μMs*^−1^	[105]
**Smooth Muscle**	N/A	
**Parenchyma**	$P_{NO}-k_{O_{2}}[O_{2}][NO][Cell]$	[77]
**Constants**	Value	
***Hct* (hematocrit)**	0.45	*text*
***Cell* (cell density)**	10^8^ *cells ml*^−1^	[124]
**[*Hb*_*RBC*_] (hemoglobin concentration in red blood cells)**	20.3 *mM*	*Text*
**[*Hb*_*CFL*_] (hemoglobin concentration in cell-free layer)**	1 *μM*, 20 *μM*, or 40 *μM*	*text*
***k*_*RBC Hb*_ (reaction constant of Hb with NO in RBCs)**	1.4∙10^5^ *M*^−1^*s*^−1^	[82,125]
***k*_*CFL Hb*_ (reaction constant of Hb with NO in the cell-free layer)**	5.8∙10^7^ *M*^−1^*s*^−1^	[126]
**$k_{O_{2}}$ (O_2_ dependent reaction constant NO in the parenchyma)**	$5.38∙10^{-4}M^{-1}s^{-1}{\left[\frac{cell}{ml}\right]}^{-1}$	[77]
**ε (permeability of oxygen)**	1.39 μM mmHg^-1^	[127,128]
**D_NO_ (diffusion coefficient of NO)**	3300 μm^2^ s^-1^	[129]
**D_O2_ (diffusion coefficient of O_2_)**	4000 μm^2^ s^-1^	[127,128]
**P_O2 artery_ (partial pressure of O_2_ in the artery)**	65 mmHg	[130–133]
**ρ (cellular metabolic rate of oxygen consumption)**	3 μmole cm^-3^ min^-1^	[128], [134,135]
**R_t_ (radial distance to parenchymal tissue boundary)**	100 μm	[113,136]
**EC_50_ (half-maximal excitatory concentration of NO for GC**)	8.9 nM	[51,117,118]
**n (Hill coefficient)**	0.8	[51,117]
**ζ_O2_ (Km value of O_2_ for the CcO)**	210	[56,137,138]
**ζ_NO_ (Km value of NO for the CcO)**	0.225	[56,137,138]
***Hct*_*cap*_ (capillary hematocrit)**	0.23	[139]
**f_c_ (fraction of parenchyma composed of small vessel)**	0.01	[140]
**μ_*RBC core*_ (viscosity in the red blood cell core)**	7 cP	[141,142]
**μ_*CFL*_ (viscosity of plasma)**	1.2 cP	[141]
***m* (arteriole sensitivity to NO)**	1–5 $\frac{\Delta diameter,\;\%}{\Delta GC\;activation,\;\%}$	

### Impact of blood flow on NO concentration in the smooth muscle

We first asked what impact the flow of blood through the arteriole had on the concentration of NO in the smooth muscle (**S1 Fig**), as the flow of blood will tend to move NO downstream. We simulated both a Poiseuille flow profile and the more blunted flow profile in a 20 μm diameter arteriole (see Methods). We varied the flow over a wide range of velocities, spanning the entire physiological range (centerline velocities < 20 mm/sec) well into the physiologically implausible range (up to 150 mm/sec). The GC activation (measured at the axial midpoint of our 400μm long model) was 0.1% lower in the model with the highest physiologically plausible levels of flow than with the model with no convection or flow. To put this in perspective, this change in GC activation will cause a 0.1 to 0.5% change in vessel diameter in our model (see Methods). The blood entering the model has a NO concentration of 0 mM, so we wanted to know over what distance the NO equilibrated in the smooth muscle. To address this, we plotted NO concentration in the model as a function of radial and axial distances (**S2 Fig**). The NO concentration reaches equilibrium in the smooth muscle within ~25 μm of the entry of the blood, which is very small relative to the extent of a penetrating arteriole (>1mm). The expected half-life of NO in the blood is less than 2 milliseconds [143] during which NO would travel 24 μm in blood traveling at 12 mm/s. Thus, convectional transport of NO signals in the blood is limited in the axial direction. Because we did not assume that there was any variation in NO production along the axial direction, convection did not play an appreciable role in determining the NO concentration in the smooth muscle. We also tested how transient changes in flow velocities affected NO concentration in smooth muscle and found similar small effects (**S3 Fig**). These simulations are consistent with previous work that has suggested that convection by blood has a small effect on NO concentration in the peripheral vasculature [79,104,144]. As the effects of convection by the blood on NO levels were relatively small, in all subsequent simulations we did not include convective effects.

### Effects of vessel size and NO production location on smooth muscle NO concentration

We then asked how the spatial arrangement of NO production relative to the arteriole and the size of the arteriole impacted the concentration of NO in the smooth muscle. We explored three different spatial profiles of NO production (**Fig 2A**). Early models of NO diffusion dynamics assumed homogenous NO production in the parenchyma, which we refer to as the ‘uniform’ condition. However, there is anatomical evidence that nNOS-expressing neurons and their processes may be concentrated around arterioles [33,122,123] (**Fig 2A**, proximal). In the proximal geometry, all NO was produced within 2 μm of the smooth muscle [123]. We also considered an intermediate case, which we refer to as the ‘regional’ geometry. In this case, NO production is higher within 50 μm of the vessel [33]. In the uniform case, NO is produced uniformly throughout the parenchyma. We emphasize that we do not mean for these geometries to be detailed reconstructions of the actual NO production, but rather exemplars that allow us to understand the role of the spatial distribution of NO production in neurovascular coupling.‬‬‬‬‬‬‬‬‬‬‬‬‬‬‬‬‬‬‬‬ We parametrically varied NO signaling for each combination of arteriole diameter, NO production and NO production location (**Fig 2D**) and evaluated their ability to signal the arteriole by the effective activation of guanylyl cyclase (GC) in the smooth muscle (**Fig 2C**). To match a given concentration of NO in the smooth muscle for a given geometry, the rate of NO production was varied. This is shown in **Fig 2B**, where the NO production rate (averaged over the entire parenchymal region) to reach 50% of the maximal activation of GC in a 40 μm diameter arteriole (outlined with a box in **Fig 2D**) was 0.02 M/s for the proximal geometry, 0.05 M/s for the regional geometry and 0.056 M/s for the uniform geometry.

We found that when holding the rate of NO production constant, the size of the vessel had an impact on the concentration in the smooth muscle. This can be seen by the upwardly sloping contour lines in all of the NO production geometries (**Fig 2D**). If there was no size dependence, these contour lines would be horizontal. This size dependence was due to the higher degradation rate of NO in the hemoglobin rich portion of the blood relative to the degradation rate in the tissue. As arteriole diameter increases, more hemoglobin is present and more NO will be removed such that a higher production rate of NO is required to maintain the same concentration of NO in the smooth muscle. This parallels the experimental observation that the dilation of a vessel, as measured as a percentage of its baseline diameter, is inversely related to its resting size [1,145,146], suggesting that degradation of NO by hemoglobin may contribute to the size-dependence of arteriole reactivity. A vessel with a larger resting diameter will degrade more NO than a smaller diameter vessel, resulting in smaller increases in smooth muscle NO and consequently a smaller vasodilation. We explored the impact of vessel-size dependence on NO degradation in our dynamical models of dilation below.

We also find for a given concentration of NO in the smooth muscle, the different NO production geometries show markedly different concentrations of NO in the parenchyma (**Fig 2B**). This is because NO is not only degraded in the blood, but also in the tissue (albeit at a substantially lower rate). The further the NO must diffuse to reach the smooth muscle, the larger the fraction of NO that will be degraded before reaching its target. This means that the concentration of NO at a distant source (the parenchyma in the uniform model) must be higher than for a closer source (the proximal model). This high concentration of NO in the brain tissue for the uniform and regional production models can have adverse effects on mitochondrial respiration when oxygen levels fall, which we explore below.

### Impact of NO levels on mitochondrial respiration

We set out to determine the impact the spatial pattern of NO production has on mitochondrial respiration. High levels of NO are toxic, because NO competes with oxygen for the rate-limiting enzyme in aerobic respiration, cytochrome c oxidase [57,147]. At very high levels of NO and low levels of oxygen, the reaction of NO with cytochrome c oxidase can be irreversible [148], though there is evidence that this inhibition will not be irreversible at concentrations closer to physiological values [149]. The inhibition of mitochondrial respiration by NO puts an upper limit on the levels of NO present in the healthy brain. Using the NO concentration profiles calculated above, combined with peri-arterial oxygen profiles derived from *in vivo* oxygen measurements using phosphorescent oxygen probes [130–133], we calculated the cytochrome c oxidase activity as a function of distance from the simulated penetrating arteriole (**Fig 3A**). Close to the artery, the capillary density is low, and oxygenation of tissue is largely supplied by the artery [150]. As respiration depends on oxygen levels, the respiration rate will fall with distance from the vessel. However, this only becomes an issue for regional and uniform models of NO production. At levels of NO production that drive high levels of guanylyl cyclase activation in the smooth muscle, the combination of high NO levels and low levels of oxygen will result in substantial inhibition of mitochondrial respiration in the tissue distant from the vessel. A parameter sweep of NO production rates (expressed as guanylyl cyclase activation in the smooth muscle) and vessel size shows that for both the regional and uniform models, high levels of NO production reduce the CcO activity below 12.5% of normal for an appreciable fraction of the tissue (**Fig 3B**). The potential toxicity of high NO concentrations remained even when modeling NO scavenging by hemoglobin in capillaries by increasing the degradation rate in the parenchyma (**S4 Fig, S5 Fig**). Though this hypoxia will be mitigated by capillaries supplying oxygen to tissue distant from the arteriole, these simulations suggests that keeping the site of NO production close to the smooth muscle may prevent tissue damage associated with high NO levels.

### Biphasic hemodynamic responses from increased NO removal by blood during vasodilation

**Fig 4:**
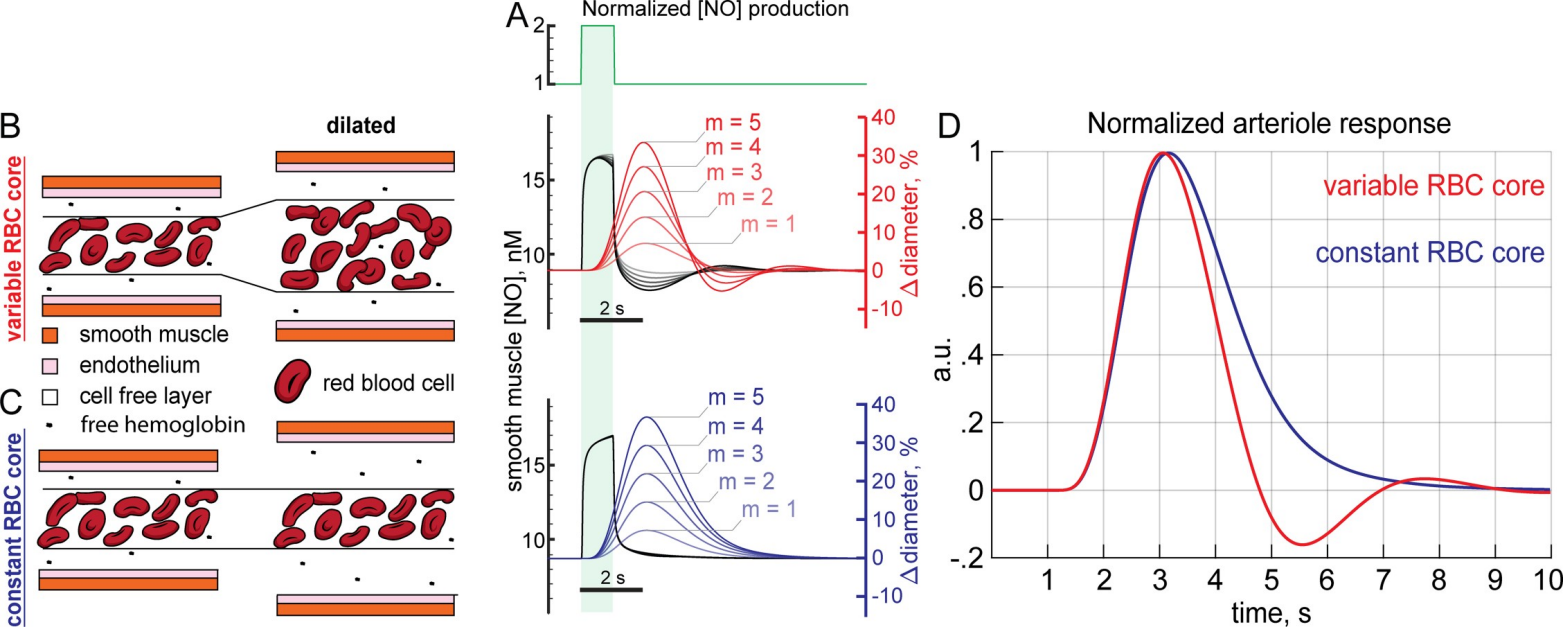
Dynamical model of NO-induced dilation shows a post-stimulus undershoot. All simulations for this figure used a dynamic model with a baseline diameter of 20 μm. A) NO production in the proximal model was increased 100% for 1 second in these simulations with a baseline NO production set at EC_50_ (the concentration of NO that produces half maximal activation of GC) in the smooth muscle. B) Arteriole dilation increases the supply of RBCs in the variable RBC model, where the RBC core changes diameter with vessel diameter. The arteriole dilates (red) in response to increased NO in the smooth muscle (black); however, after NO production returns to baseline the arteriole is still dilated. The dilated arteriole can accommodate more RBCs which depletes NO below baseline. The depletion of NO concentrations below baseline is reflected in a corresponding post-stimulus constriction. Five different sensitivities to GC (m = 1,2,3,4,5 $\frac{\Delta diameter,\;\%}{\Delta GC\;activation,\;\%}$) are shown. C) Dilation in the constant RBC core case does not increase RBC supply or the degradation rate of NO. Note that fixed core model is not realistic. When vasodilation does not increase NO consumption, NO concentrations do not fall below baseline and no post-stimulus constriction occurs. D) Dilations for m = 5 $\frac{\Delta diameter,\;\%}{\Delta GC\;activation,\;\%}$ rescaled to the same height showing the relative size of the post-stimulus constriction in the variable RBC core case while none is present if the RBC core is held constant during vasodilation.

A larger arteriole degrades more NO than a smaller one, enough to alter NO levels appreciably in smooth muscle at steady state. We then investigated whether a similar process could occur during vasodilation and what impact it would have on vasodynamics. We moved to a dynamic model, in which the concentration of NO in smooth muscle dynamically dilated the vessel (**S6 Fig**). An important parameter in these simulations is the sensitivity of the dilation to changes in GC activation, captured in our simulations in the parameter ‘m’, (which has units of $\frac{\Delta diameter,\;\%}{\Delta GC\;activation,\;\%}$, see Methods). The sensitivity of arteries to NO is known to vary [51,119,151–153], and the larger m is, the more sensitive the artery is to changes in NO concentrations. Empirically, studies suggest m is in the range of 1–5, with m = 5 giving dilations comparable to the largest stimulus evoked dilations in awake animals [1,146,154,155]. The key interaction in this model was that the dilation of the arteriole caused an increase in the local hemoglobin concentration via an increase in the size of the red blood cell-containing ‘core’ (RBC core) (**S6 Fig, Fig 4B**). This increase in hemoglobin would in turn cause an increase in NO degradation, which functions as a delayed negative feedback on NO levels in the smooth muscle. The dilation will be delayed relative to the increase in NO production due to diffusion time, and the latency of the signal transduction cascade transducing the elevation of NO levels in the smooth muscle into relaxation. We wanted to understand if this separation of timescales could produce vasodynamics like those seen *in vivo*. Because the proximal model minimizes potential CcO inhibition and previous work points to NO sources close to the arteriole [33,122,123] we use the proximal NO production geometry for all subsequent simulations.

We first simulated the effects of a transient increase in NO production, similar to what would be generated in response to a brief elevation of neural activity in response to a stimulus. The effects of a stimulus were implemented by doubling parenchymal NO production for 1 second (**Fig 4A**). When the increased arteriole diameter elevated NO scavenging by increasing the amount of hemoglobin (**Fig 4B**), NO concentrations in the smooth muscle dropped below baseline during vasodilation (**Fig 4B,** black), even though there is no corresponding decrease of NO production below baseline (**Fig 4A**). The drop in NO concentration in the smooth muscle results in a post-stimulus undershoot (**Fig 4B,** red), reminiscent of the canonical hemodynamic response function (HRF) seen *in vivo* [1,29,156]. We hypothesized that this undershoot was driven by the increased hemoglobin in the vessel that would naturally take place when the vessel dilated. To test this hypothesis, we performed simulations where the RBC core was kept at a constant diameter when the arteriole dilated (**Fig 4C**). Without the increase in NO degradation mediated by an increase in hemoglobin, the post-stimulus undershoot was not observed (**Fig 4C,** blue). To better visualize the differences between the two conditions, we plotted the two responses together (**Fig 4D**). The (physically realistic) variable core model shows a clear undershoot, (**Fig 4D,** red) while the constant core model does not (**Fig 4D,** blue). The variable core model could generate arterial dilation dynamics qualitatively similar to those seen in awake mice in response to sensory stimulation [1] (**S7 Fig**). While dilation was linear with m, the undershoot was not, as it was only present when m>2. Together, these suggest that the increased NO scavenging in the RBC core during vasodilation can be a contributing factor to the post-stimulus undershoot in arterial diameter.

### Interplay of vasodilation and NO degradation can generate vasomotion oscillations

**Fig 5:**
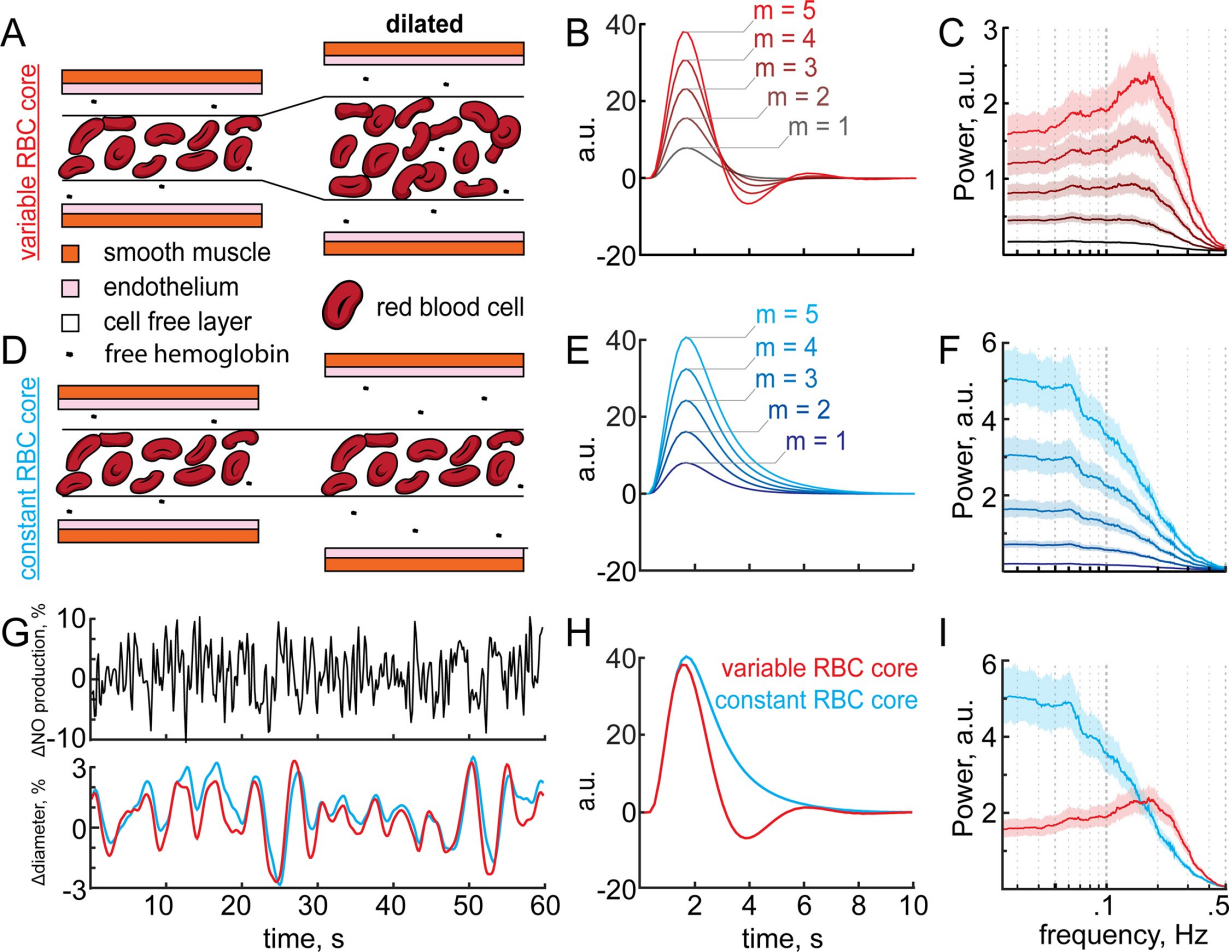
Arteriole sensitivity to NO increases the amplitude of the undershoot and vasomotion. All simulations for this figure used a dynamic model with a baseline diameter of 20 μm. A) Schematic of the variable RBC core model. Vasodilation increases the diameter of the RBC core and thus the degradation rate of NO in the variable RBC core model. B & C) Effective hemodynamic response function (B) and power spectrum (C) of the variable RBC core model from a white noise NO production rate. Note that with increasing NO sensitivity (slope, m $\frac{\Delta diameter,\;\%}{\Delta GC\;activation,\;\%}$), the magnitude of the undershoot and power near 0.2 Hz increases. D) Constant RBC core model where vasodilation does not increase the diameter of the RBC core and thus the degradation rate of NO does not change. E & F) No post-stimulus undershoot is present (E) and the maximum power of the constant RBC core model is at low frequencies (<0.1 Hz) (F). G) 60 second example taken from a 25 minute trial displaying NO production and resultant diameter changes from which arteriole dynamics were evaluated. H & I) Juxtaposition of variable (red) and constant (blue) RBC core models for m = 5 showing the post-stimulus constriction (H) and peak power between 0.1–0.3 Hz (I) in the variable RBC core case (red) while no undershoot or peak power between 0.1–0.3 Hz is present if the RBC is held constant during vasodilation (blue).

We then sought to quantify the effects of an increase in NO scavenging accompanying dilation on arteriole dynamics. The relationship between a stimulus or neural activity and the change in vessel diameter is captured by the hemodynamic response function [156] (HRF). The HRF is a linear kernel that low-pass filters neural activity into a change in vessel diameter. This kernel can be easily extracted from the response (in this case, artery diameter) to a spectrally white input [146,157,158] (in this case, NO production linked to neural activity). To better understand how NO degradation dynamics impact neurovascular coupling, we simulated the responses of both the variable RBC core and constant RBC core models (**Fig 5G,** red & blue) to randomly varying (‘white noise’) NO production (**Fig 5G**, black). We then deconvolved out the *effective* HRF of the models (using the modified Toeplitz matrix method [156]) (**Fig 5B and 5E**) from the vascular response. Note that the effective HRF is empirically determined from our simulations, and can differ in shape from the h(t) function used to relate NO concentration to vessel diameter (see Methods) if the changes in vessel diameter alter NO concentration in the smooth muscle. We found that there was an undershoot in the effective HRF of the variable RBC core model (**Fig 5B**), but no undershoot following the dilation in the effective HRF of the constant RBC core model (**Fig 5E**). The undershoot was driven by the decreased NO levels in the smooth muscle accompanying dilation due to the larger amount of hemoglobin in the dilated artery (**Fig 4B**), and the magnitude of the undershoot increased with increasing sensitivity to NO (**Fig 5B**). Even though the undershoot was an emergent property in the simulations, it was still linear, as the variance explained by the effective HRF was very high (R^2^~0.95) (**S8 Fig**). By looking at the power spectrum of the arteriole diameter changes elicited by white noise NO production we can see the frequency response properties of the system. Interestingly, the power is highest in the 0.1–0.3 Hz frequency band of the power spectrum of the artery diameter in the variable RBC core model (**Fig 5C**), showing that this system effectively acts as a band pass filter. This peak is reminiscent of vasomotion, a rhythmic 0.1–0.3 Hz oscillation in cerebral artery diameter observed in awake and anesthetized animals, *in vitro* and in humans [17–19,156,159,160]. When the vasodilation does not increase NO scavenging, as is the case when the RBC core is held constant, no undershoot (**Fig 5E**) or elevation of power in the 0.1–0.3 Hz band were observed (**Fig 5F**). The amplitude of the post-stimulus constriction and power of vasomotion-like oscillations were enhanced in arterioles sensitive to NO (m = 5), but minimized or absent in arterioles with lower NO sensitivity (m = 1) suggesting that arteriole sensitivity to NO is an important factor in the emergence of these arteriole dynamics (**Fig 5B and 5C**). This comparison of the dynamic and constant RBC core models highlights the importance of NO degradation on vascular dynamics (**Fig 5H and 5I**). These dynamics were a result of NO diffusion and degradation dynamics as the imposed vascular response to NO alone was not able to reproduce these dynamics. The effects of NO scavenging by increased hemoglobin likely work in concert with other drivers of vasomotion [161–164] to generate these oscillation *in vivo*.

One concern is that the observed dynamics (post-stimulus undershoot and band-pass properties in the 0.1–0.3 Hz range) are due to our choice of HRF. This appears to not be the case, as when the the HRF function alone is convolved with white noise or an impulse response function, there is no peak in the 0.1–0.3 Hz range, or post stimulus undershoot (**S9 Fig**), and the system displays pure low-pass behavior. Furthermore, when we slightly slowed the dynamics of the effective HRF in the dynamic model (**S10 Fig**) so that it peaked ~2.1 seconds post-stimulus rather than 1.5, we observed a *larger* post-stimulus undershoot and a *larger* peak in the 0.1–0.3 Hz band. These results show that the post-stimulus undershoot and vasomotion-like oscillations we observed in our model are not a simple consequence of our choice of HRF but require the feedback due to increased NO consumption during dilation to emerge.

### Influence of plasma free hemoglobin and hematocrit on vasodynamics

Because NO is mainly degraded by the blood, we expected that changing hematologic properties such as free hemoglobin (Hgb) or hematocrit (Hct) would alter NO-mediated signaling. Hematocrit varies with sex [165], and can be elevated by drugs [89,166] or prolonged exposure to high altitude [167,168]. While NO is typically degraded by hemoglobin (Hgb) in RBCs, free Hgb in the plasma can scavenge NO up to 1,000-fold faster than within RBCs [125,143]. Under normal conditions free Hgb levels in the plasma are low, and the impact of this free Hgb on NO levels is minimal. However, plasma free Hgb can be elevated in sickle cell disease [88], malaria [169] or following blood transfusions [170]. Elevation of free plasma Hgb can cause cardiovascular issues [90,171–174] due to the increased scavenging of NO [175,176].

**Fig 6:**
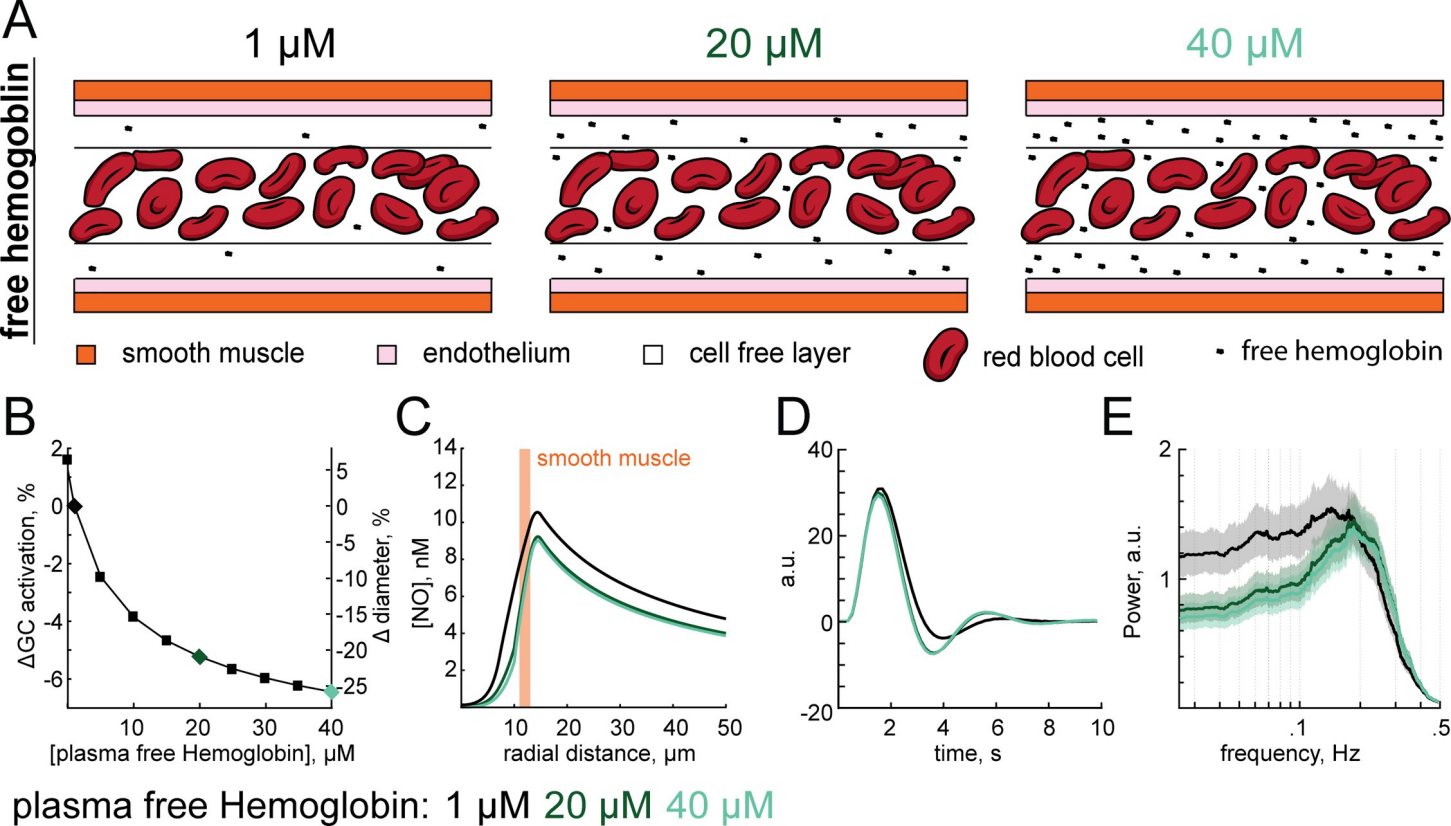
Impact of plasma free hemoglobin on vasodynamics. All simulations in this figure used a dynamic model. The baseline vessel diameter was 20 μm. A) When high levels of hemoglobin (Hgb) is present in the plasma, the location of NO degradation shifts from the RBC core to the Hgb rich cell free layer. B) Increasing free Hgb constricts arterioles. Plot showing the absolute % change in GC activation from baseline GC activation (50%) as a function of plasma free hemoglobin. For these simulations, the plasma free hemoglobin was altered, and change in diameter was calculated once the vessel diameter equilibrated. Black, dark green and light green diamonds correspond to 1, 20 and 40 μM of plasma hemoglobin respectively, with colors interpretations conserved from B to E. C) The shift in location of NO consumption to the cell free layer and increased reactivity of free Hgb over RBC Hgb decreases perivascular NO, with minimal additional effect when free Hgb was elevated past 20 μM. D) Increasing free hemoglobin slightly increases the undershoot in the hemodynamic response function. E) Increasing free hemoglobin reduces the low frequency power (<0.1 Hz) and strengthens the band pass properties within the 0.1–0.3 Hz range.

We first explored the effects of altering plasma free Hgb. Increasing free Hgb (**Fig 6A**) reduced arteriole diameter (**Fig 6B**) though depletion of perivascular NO (**Fig 6C**), consistent with *in vivo* experiments [177]. The increase in free Hgb resulted in a larger post-stimulus undershoot (**Fig 6D**) and an increase in the band-pass like properties of the arteriole (**Fig 6E**). These simulations suggest that in addition to other symptoms, elevated plasma free hemoglobin may also cause constriction of cerebral arterioles and alter the dynamics of hemodynamic responses. Increasing hematocrit resulted in decreases in baseline arteriole diameter (**S11B Fig**) and perivascular NO (**S11C Fig**) in the model. However, neither the effective HRF, nor frequency response properties of the vessel were appreciably affected by varying the hematocrit (**S11D and S11E Fig**). The lack of an effect can be attributed to the fact that even under different hematocrit concentrations the location of NO scavenging remains unchanged. However, when increasing free Hgb in the plasma, the compartmentalizing effects of the CFL is compromised, and the location of NO scavenging shifts from the center of the lumen to much closer to its source [79,102,178,179]. With NO being scavenged much closer to the smooth muscle, any changes to the rate of scavenging (such as increased hemoglobin during dilation) are amplified. While both hematocrit and free Hgb in the plasma contribute to determining baseline arteriole tone, these simulations suggest that plasma free hemoglobin can also have a substantial effect on vasodynamics through a NO-mediated mechanism.

### NO can act as sensor of cerebral oxygenation

**Fig 7:**
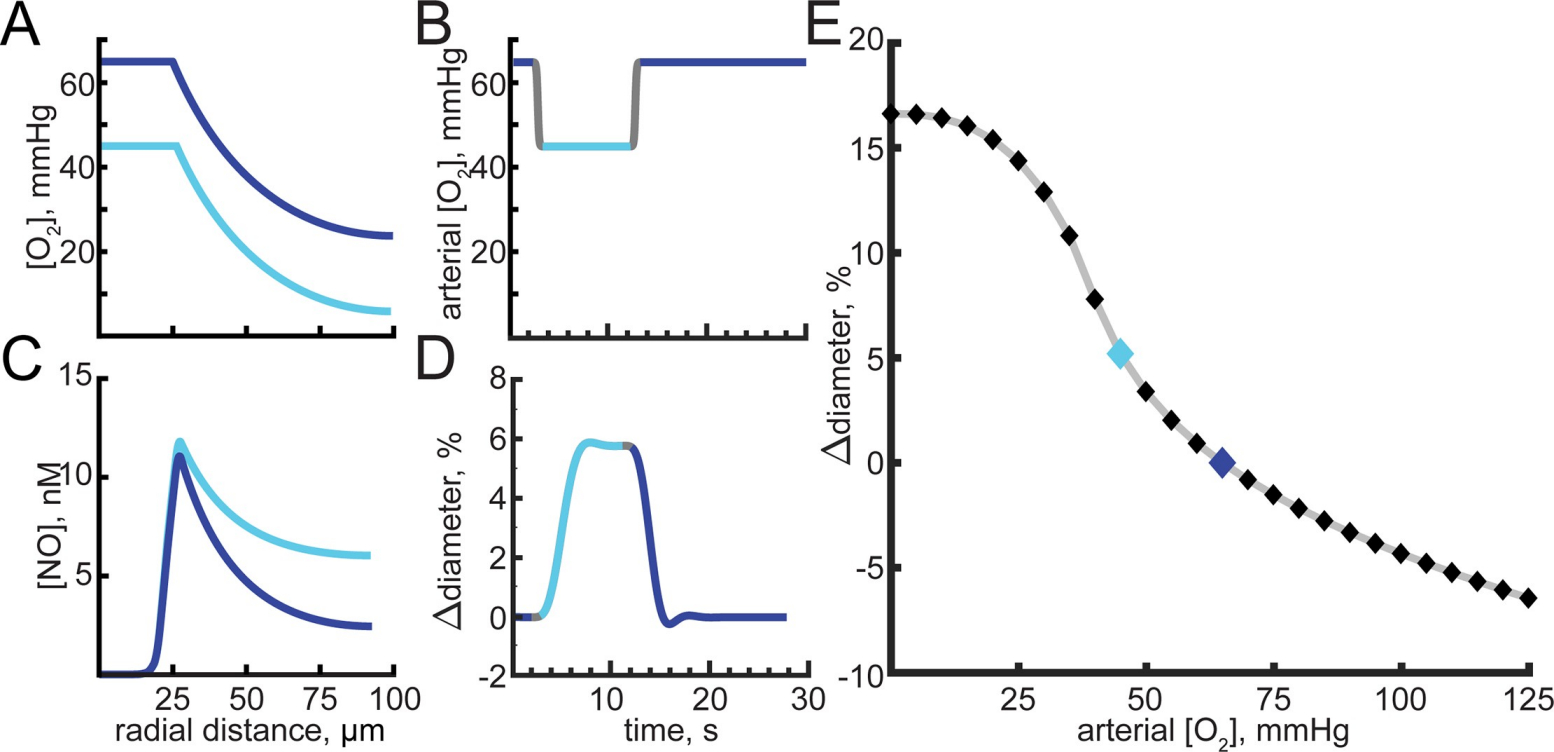
Hypoxia and hyperoxia alter NO levels and can drive vasodilation and vasoconstriction. All simulations in this figure used a dynamic model. In these simulations we varied arterial oxygen concentration, and the baseline diameter of the vessel was 50μm. A) Oxygen concentration as a function of distance from the arteriole center with a blood oxygen content of 65 mmHg (dark blue) or 45 mmHg (light blue). B) Time course of arterial oxygenation. The oxygen concentration drops 20 mmHg for 10 seconds before returning back to 65 mmHg. Gray indicates time at which arteriole oxygen levels are changing. Dark blue and light blue indicate arteriole oxygen content of 65 mmHg or 45 mmHg, respectively. C) Perivascular NO concentrations with a 65 mmHg (dark blue) and 45 mmHg (light blue) blood oxygen content. D) Arteriole response from a 10 second long 20 mmHg decrease in blood oxygenation shown in (B). Arteriole sensitivity to NO is set to m = 4. E) Hypoxia increases arteriole diameter at a more rapid rate that hyperoxia. Dark and light blue diamonds correspond to blood oxygenation states shown in (A-D).

Despite the lack of an known oxygen sensor in the brain, hypoxia will dilate and hyperoxia will constrict cerebral arterioles [180–197]. These cerebrovascular responses to blood oxygenation are modulated by NO availability [180,192,198–202], occur under isocapnic conditions [200,202] and constant pH [200]. We wanted to investigate if changes in NO consumption due to oxidative reactions in the parenchyma could contribute to hypoxia-induced vasodilation. The first order dependence of NO removal on tissue oxygen concentration [77] would mean that NO would be degraded faster under a hyperoxic condition. Elevated oxygen concentrations could constrict arterioles by depleting perivascular NO, and conversely low oxygen could dilate arterioles by consuming less NO, effectively allowing NO to functioning as a local oxygen sensor. We tested this idea by dynamically varying the oxygen levels in the artery (**Fig 7A and 7B**) and looking at the resulting changes in vessel diameter (**Fig 7D**) due to changes in parenchymal NO degradation (**Fig 7C**). Using a baseline arteriole oxygen concentration of 65 mmHg [130–133], we varied arteriole oxygen levels in the range from 0 to 125 mmHg [203]. Arterial oxygenation dynamically tracks respiration rate [204]. Hypoxia drove dilation, and hyperoxia drove vasoconstriction, though not with as large of magnitude (**Fig 7E**). The observation that in our model hypoxia drove a larger dilation than hyperoxia drove constriction is consistent with *in vivo* observations [189,193,205] that the magnitude of the increases in cerebral blood flow caused by hypoxia is larger than the magnitude of the decrease in cerebral blood flow caused by hyperoxia. These results highlight NO’s potential to function as a local oxygen sensor by linking perivascular oxygen concentrations to vascular tone through an oxygen dependent rate of NO removal in the parenchyma.

### Impact of vasodilation on parenchymal NO concentration

**Fig 8:**
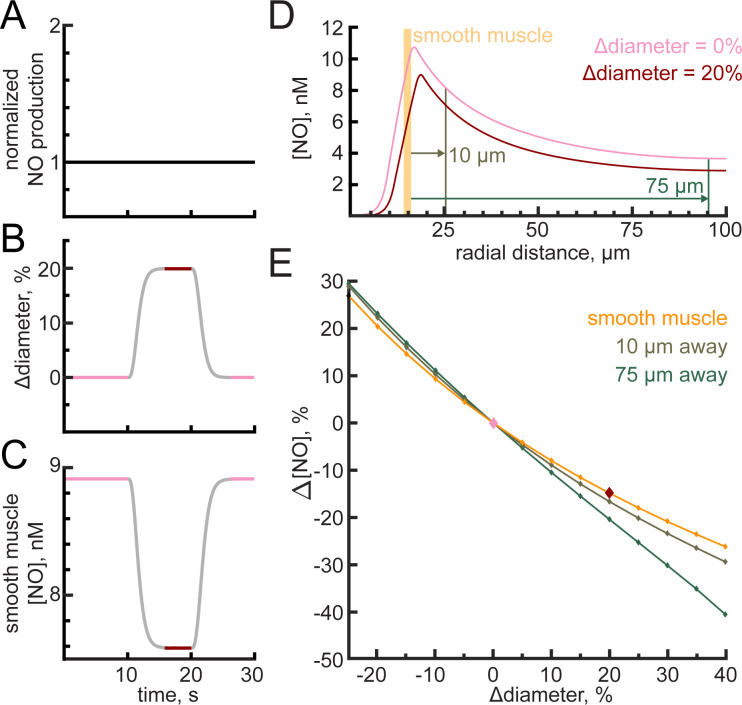
Arteriole diameter changes alter perivascular NO. The dilation in the simulations in this figure was imposed and was uncoupled from the NO concentration in the smooth muscle. The baseline diameter of the vessel was 25 μm. A) NO production was held constant. (B) A 20% dilation of the arteriole was externally imposed. Pink indicates the pre-dilated state while red is the dilated state. Grey indicates transition times in which steady state has not yet been reached. C) NO concentrations in the smooth muscle depleted as a result of the increased arteriole diameter. D) The perivascular NO in the smooth muscle (orange), 10 μm from the arteriole wall (brown), and 75 μm from the arteriole wall (green) were all decreased following a 20% dilation. The reference position of the smooth muscle is shown in orange, and parenchymal position markers in brown and green is for the pre-dilated state. E) Perivascular NO changed during arteriole dilation and constriction even at distances up to 75 μm from the arteriole wall.

It has been proposed that changes in the vasculature can drive changes in neural activity [106]. As the degradation rate of NO is greatly influenced by the amount of hemoglobin and NO levels affect neural excitability [73,74], we hypothesized that changes in NO concentration driven by vasodilation might be able to drive changes in NO levels of nearby neurons. In all our previous simulations, the concentration of NO in the smooth muscle has thus far changed with size of the arteriole. Here we asked how vasodilation due to other pathways [2,8,12,24–27,31,33,206] will impact parenchymal NO levels. We isolated the influence of vasodilation on parenchymal NO in the model by imposing changes in arteriole diameter (**Fig 8B**) in the background of a constant NO production rate (**Fig 8A**). This vasodilation caused a decrease of NO in the smooth muscle (**Fig 8C**). Because as the vessel dilates, it slightly distorts the tissue, we looked at the parenchymal NO concentrations relative to the outer edge of the smooth muscle (adjusted for deformation), rather than from the vessel centerline. We found that vasodilation caused an appreciable drop in the NO concentrations in the parenchyma (**Fig 8D**). We then parametrically varied the sign and amplitude of the vessel diameter change and looked at the impact of these diameter changes on parenchymal NO levels. We found that dilation and constriction in a physiologically plausible range can produce changes in parenchymal NO of approximately 10% (**Fig 8E**). To our knowledge, there are no quantitative measure of how GC activation can affect neural excitability, but this magnitude of a change could be large enough to alter neural excitability. These simulations identify a potential mechanism by which neurons can receive information about the state of nearby vessels.

### Sensitivity of the results to model assumptions

In order to understand how the results of the model depend on various assumptions, we performed simulations where we altered the EC_50_ of GC and the basal GC activation (**S12 Fig**), increased parenchymal degradation of NO to simulate the effects of capillaries and varied the spatial pattern of NO production (**S13 Fig**). In general, we continued to observe a post-stimulus undershoot and vasomotion-like 0.1–0.3 Hz oscillations under all of these conditions, with the notable exception of when the baseline GC activation was low.

## Discussion

### Limitations

There are many limitations to our model that should be kept in mind. Our model is by necessity a simplification of NO signaling pathways and of the complex interaction between the vasculature and the brain. For example, we assumed a linear relationship between GC activity in the smooth muscle and the diameter, and this relationship is unlikely to be so simple in reality. We did not simulate other neurovascular coupling pathways [2,8,12,24–27,31,33,206]. This should not be taken to mean that NO-mediated coupling is the only (or even primary) neurovascular coupling mechanism, as NO-mediated dilation acts on other pathways, such as the arachidonic acid signaling pathway. We also chose our HRF function (h(t)) to match the dynamics of experimentally observed dilations in vivo, and many aspects of the observed response, such as the onset dynamics of dilation, depend on the details of this function. The transfer function relating NO exposure to vasodilation, as well as the sensitivity of an arteriole to NO (m) was also taken to be time-invariant during the simulation; however, the dynamics of GC deactivation are slower than GC activation [207–209] and this is likely to produce long lasting effects that will not be captured by our model. Also, GC activation can occur at lower NO concentrations [210,211] or independently of NO signaling [212] which may play a role in other biological processes [213]. We did not model GC activation dynamics or the mechanical properties of the arteriole to obtain vascular responses but fitted the positive component of the transfer function to an empirically determined kernel from in vivo measurements [1,156]. Vasomotion in our simulations is due to increased degradation of NO with vasodilation, which acts as negative feedback on the vessel diameter. As with neurovascular coupling, NO is not the only driver of vasomotion, there is good evidence for other, non-exclusive mechanisms of vasomotion [164] that the mechanism proposed here can work with. The parameters for the cell free layer are taken from steady-state measurements, and these may not hold true during dynamic diameter changes. In our model during dilation events, hematocrit in the red blood cell core does not change as a function of the arteriole diameter in the model. However, the cell free layer does increase in thickness, and thus when considering the entire arteriole, hematocrit decreases during dilation. This leads to a conservative estimate of the influence of dilation on NO consumption as keeping hematocrit constant during dilation would provide more hemoglobin and NO scavenging during dilation events. We did not include the potential different NO reaction rates with oxygenated and deoxygenated hemoglobin [214] (but see [215–217]) or any oxygen-dependence of NO synthesis [218]. However, hypoxia causes an increase in tissue NO levels [219] and blocking NOS blocks hypoxia-generated dilations [220], so oxygen-dependent degradation of NO is likely to dominate physiologically. We also did not model changes in eNOS activity, as previous work has shown that the dilation and flow changes accompanying functional hyperemia do not cause significant changes in shear stress in arterioles [221], though changes in eNOS activity could also play a role. We did not vary NO production in the axial direction and thus the influence of convection was minimal. Finally, we used simplified vascular and neural geometries in order to gain insight into how NO production and degradation dynamics might impact neurovascular coupling. Arterioles are not symmetrical, nor would all sources of parenchymal NO be adjacent to the vessel wall. Future work that combines large-scale vascular reconstructions [113,114] paired with detailed mapping of neuronal cell-type locations [140,222] in the brain will allow the creation of more realistic NO diffusion models that may give insight into the heterogeneity of neurovascular coupling across brain-regions [223–226].

### Robustness of modeling results

While there is a wide range of NO concentrations measured in the tissue [56], the levels of NO that have been shown to drive GC activation have been consistent across several studies [51,117]. We manipulated NO production rates over a wide range (**Fig 2**) and found that (at steady state), for a given NO production rate, the larger the vessel the lower NO concentration in the smooth muscle (expressed as GC activation) due to the degradation by hemoglobin. This shows that at least at steady state, the amount of hemoglobin (which is controlled by the vessel diameter) plays an important role in the NO levels in the smooth muscle, no matter what the NO production level (and thus concentration) was. We also varied the dynamics of the HRF (**S10 Fig**) and found that so long as the artery did not dilate unphysiologically quickly, we saw a post-stimulus undershoot and vasomotion-like oscillations. This behavior also persisted when we used a 50% lower EC_50_ value (**S12 Fig**) but was not as pronounced when the basal activity of GC was lower (**S12 Fig**), suggesting that a baseline level of GC activation is required for the effects observed here. These dynamics were also present when accounting for capillary consumption of NO or with increasing background NO production by the parenchyma (**S13 Fig**). While we did not test whether these results held true for every possible combinations HRF, NO production level, baseline GC activation level, etc. in dynamic models, we found that for the range of parameters we used, both the post-stimulus undershoot and vasomotion were present. Additionally for the model parameters tested (vessel sensitivity (m) (**Fig 5**), EC_50_ (**S12 Fig**), baseline GC activity (**S12 Fig**), presence of capillaries (**S13 Fig**), HRF shape (**S10 Fig**), hemoglobin (**Fig 6**), hematocrit (**S11 Fig**), and increased background NO production (**S13 Fig**)), we did not see changes in the frequency of vasomotion, although many of these could influence the power in the vasomotion band. This suggests that the frequency of these vascular oscillations is robustly generated by the interactions of several different factors. In total, our simulations suggest that so long as there is substantial baseline GC activation and the HRF generates dilations with realistic dynamics, NO degradation by the blood can cause a post-stimulus undershoot and vasomotion-like oscillations.

### Implications of model

Computational models of diffusion have complemented experimental techniques to give us insights into how NO signals to the vasculature. Pioneering work by Lancaster [39], Wood and Garthwaite [40], and others [60,80,96,101,102,104,105,201,227] have demonstrated the importance of NO degradation by the blood in shaping the efficacy of NO signaling. Building upon their work, we apply the insights gained from modeling NO dynamics to neurovascular coupling. By coupling NO-dependent changes in arteriole tone and blood supply to a model of NO diffusion we are able to reproduce many of the commonly observed dynamics in the cerebral vasculature. These include the size dependence of arteriole dilation, vasomotion, the post-stimulus undershoot, and hypoxia-induced vasodilation. We show that in addition to the neural production of NO, consumption of NO by the blood also has the potential to modulate the hemodynamic response and that many pathologically homologous conditions may disrupt neurovascular coupling via increased NO degradation.

Our simulations show that NO degradation dynamics by the blood can provide a mechanism for many experimental observations of cerebrovascular dynamics [17–19,156,159,160,177,189,193,205,228]. The combination of genetically-encoded cGMP sensors [229,230] combined with optogenetic stimulation of nNOS-expressing neurons [70] should allow the ideas presented here to be examined experimentally. Importantly, our simulations also suggest that hemodynamic signals in the brain do not solely depend on neural activity, but rather can be greatly modulated by normal and pathological variations in the composition of blood.

## Methods

Simulations were performed in COMSOL (COMSOL Multiphysics: partial differential equations (pde) module version 5.2, Burlington MA), with a LiveLink to Matlab (version R2018b, Mathworks, Natick MA) to provide control of dynamic variables. Simulation outputs were analyzed in Matlab. A 400 μm long penetrating arteriole was modeled in the center of a 100 μm radius cylinder of parenchymal tissue with a zero-flux boundary condition. Calculations were simplified by taking advantage of radial symmetry and assuming no concentration gradients in the circumferential (θ) direction. Axial gradients of NO did not play a role unless convection was considered. All domains were assumed to have homogenous properties.

### Overview of model formulation and governing equations

In addition to diffusive movement of NO, the flow of blood could add a convective component to the movement of NO. To determine whether convection of NO driven by the flow of blood plays an appreciable role in NO dynamics, we simulated fluid flow in the vessel lumen in a full 3-D model and examined the impact of transport on NO concentration in the smooth muscle (**S1 Fig**). Note that the COMSOL files also contain the ability to include convective flow calculations by specifying a non-zero pressure difference if desired (parameter: press1 [Torr]) (see data availability).

To investigate how NO scavenging by the blood affects hemodynamic responses we modeled the interaction at the level of a penetrating arteriole supplying blood to a region of the parenchyma (**Fig 1**). NO production rates in the parenchyma and degradation rates in the blood (**Table 1**) were used in a diffusion model to predict the hemodynamic response using the quantity of NO reaching the smooth muscle. We generated a finite element model with this cylindrical geometry in COMSOL. The finite element model was divided into five domains: a red blood cell-containing ‘core’ (RBC core), a cell free layer, an endothelial cell layer, a smooth muscle layer, and parenchymal tissue. Each domain had their respective rate of production or degradation of NO (**Table 1**). NO was free to diffuse according to Fick’s law.

$$\frac{d[NO]}{dt}=D_{NO}\nabla^{2}[NO]+R_{x}(t)$$ Eq 1

Where [NO] is the concentration of NO at any given point in space, D_NO_ is the diffusion coefficient of NO (3300 μm^2^ s^-1^) [129], and R_x_(t) is the time dependent degradation or production rate of NO unique to each domain (**Fig 1D, Table 1**). While there is disagreement as to the levels of NO in the brain [56], the NO concentration dependence of guanylyl cyclase activity is relatively well characterized [68,117] and can be used to estimate the extent of vasodilation (see Methods: Smooth Muscle).

Perivascular oxygenation was estimated using the Krogh model or Fick’s diffusion equation with oxygen. Luminal oxygen concentration was set to 65 mmHg [130–133] and oxygen in the parenchymal tissue set to have a lower bound of 10 mmHg [130]. The Krogh model is a solution to radially symmetric oxygen diffusion from a cylinder (blood vessel) at steady state [231], and is given by the equation:

$$P_{{[O}_{2}]}(r)=P_{{[O}_{2}\;artery]}+\frac{\rho }{4\varepsilon D_{O_{2}}}(r^{2}-R^{2})-\frac{\rho }{2\varepsilon D_{O_{2}}}{R_{t}}^{2}ln(\frac{r}{R})$$ Eq 2

Where P_[O2 artery]_ is arteriole oxygen content in mmHg, D_O2_ is the diffusion coefficient of oxygen in water (4000 μm^2^ s^-1^) [127,128], r is the distance from the arteriole, R is the radius of the arteriole, R_t_ is the diameter of the tissue cylinder (100 μm), ε is the tissue oxygen permeability (ε = 1.39 μM mmHg^-1^), and ρ is the cellular metabolic rate of oxygen consumption (CMRO_2_) in the parenchyma, taken to be 3 μmole cm^-3^ min^-1^, as CMRO_2_ in the awake state is double that under anesthesia [128,134,135]. For simulations where oxygen levels change rapidly (**Fig 7**), we explicitly modelled the diffusion of oxygen from the vessel lumen into the parenchyma with Fick’s equation:

$$\frac{d[O_{2}]}{dt}=D_{O_{2}}\nabla^{2}[O_{2}]–\rho$$ Eq 3

Where [O_2_] is the concentration of oxygen at any given point in space. The average distance to the nearest penetrating artery from any point in the parenchyma is of order 100 μm [113,136], so we modeled NO and oxygen diffusion into the parenchyma up to 100 μm from the arteriole with a repeating boundary condition (see Methods: Parenchyma).

### Red Blood Cell core

Red blood cells (RBCs) are not distributed homogenously in the vessel, they cluster in the center (core) of the vessel and are excluded from the volume close to the endothelial cells [85,232,233]. NO entering the RBC core region is heavily scavenged by the hemoglobin contained in RBC. The rate of NO scavenging by the RBCs was obtained by multiplying the rate of NO and RBC hemoglobin interaction (k_RBC Hb_ = 1.4 • 10^5^ M^-1^s^-1^) [82,125] with the hemoglobin concentration in a single RBC (20.3 mM), and the core hematocrit [82] was taken to be 0.45 unless otherwise specified. Additionally, free hemoglobin in the plasma occupying the spaces between the RBCs can also contribute to NO scavenging. Free hemoglobin is limited in the plasma (~1 μM) compared to hemoglobin contained in RBCs, but has a much higher reaction rate with NO (k_CFL Hb_ = 5.8 • 10^7^ M^-1^s^-1^) [126]. The plasma component of NO degradation in the RBC core was calculated by multiplying the fraction of plasma (1-Hct) with the rate of NO and hemoglobin interaction in the plasma (k_CFL Hb_), and the concentration of hemoglobin in the plasma which is varied in the model to be 1, 20, or 40 μM. The total degradation rate of NO in the RBC core was assumed to be homogeneous, and was taken to be the sum of the scavenging from RBCs and plasma components:

$$R_{RBC\;core}={-k}_{RBC\;Hb}Hct[NO][{Hb}_{RBC}]-k_{CFL\;Hb}(1-Hct)[NO][{Hb}_{CFL}]$$ Eq 4

Detailed equations and parameters can be found in **Table 1**.

### Cell Free Layer

The cell free layer (CFL) is a layer of blood plasma between the RBC core and endothelial cells. The CFL influences NO signaling by providing a region of reduced NO degradation that increases the concentration of NO in the smooth muscle [179,233,234]. The thickness of the CFL has been empirically fit for a given vessel size and blood hematocrit [83,84]. We simplified the results of their model [84] to a parabolic fit when 45% hematocrit is assumed:

$$R_{CFL}=0.35R_{vessel}-0.0075{\left(R_{vessel}\right)}^{2}$$ Eq 5

For a given vessel diameter between 10 μm and 50 μm, the thickness of the cell free layer will follow this equation. The radius of the RBC core will be the vessel radius minus the thickness of the CFL. The scavenging rate of NO in the cell free layer is the product of the rate of NO and hemoglobin interaction in the plasma (k_CFL Hb_ = 5.8 • 10^7^ M^-1^s^-1^) [126]:

$$R_{CFL}=-k_{CFL\;Hb}[NO][{Hb}_{CFL}]$$ Eq 6

The concentration of plasma free hemoglobin ([Hb_CFL_]) in the CFL was modulated in the model to be 1, 20, or 40 μM.

### Endothelial cell layer

NO is not only produced from nNOS in the parenchyma but also from eNOS contained in endothelial cells. The contribution of NO from the endothelial cell layer is thought to be much smaller than parenchymal sources [82,235], but was still accounted for in the model by assuming a constant production rate of 55 • 10^−3^ μM s^-1^ in the 1 μm thick ring between the lumen and smooth muscle [105].

### Smooth muscle

Upon entering the smooth muscle, NO activates guanylyl cyclase (GC) to induce vasodilation via increased cGMP production [51,54,236]. The relationship between NO concentration and GC activation and subsequent vessel relaxation can be described by the Hill equation with a NO half-maximal excitatory concentration (EC_50_) between 3 and 10 nM and a Hill coefficient near 1 [51,117,118,237] (**Fig 2C**). The activity of GC_f_ as a function of the average concentration of NO in the smooth muscle, [NO] was given by:

$${GC}_{f}(\left[NO\right])=\frac{GC(\left[NO\right])}{{GC}_{max}}=\frac{{\left[NO\right]}^{n}}{{\left({EC}_{50}\right)}^{n}+{\left[NO\right]}^{n}}$$ Eq 7

For our model, we used an EC_50_ of 8.9 nM and a Hill coefficient of n = 0.8 [51,117,118], except for **S12 Fig**, where an EC_50_ of 3.9 nM and n = 2.1 were used. The sensitivity of an arteriole to NO can be modulated [118,152]. In order to account for an arteriole’s ability to become sensitized or desensitized to NO, we kept changes in vessel size relatively low (±5%) by using NO productions rates that did not produce large dilations when investigating vasodynamic properties and assumed a linear relationship between GC activation and vasodilation within this range. The slope of the relationship between GC activation and vasodilation was denoted by the variable $m\left(\frac{\Delta diameter,\;\%}{\Delta GC\;activation,\;\%}\right)$ which was varied between 1 and 5 in our model.

The dilatory response following brief sensory stimuli usually peaks after 1–2 seconds [1,156,158,238], and can be mathematically described by the convolution of the hemodynamic response function (HRF) with the stimulus. The HRF is typically modeled by fitting with a gamma distribution function [15,239]. In some cases, in order to capture the post-stimulus undershoot the HRF is modeled as a sum of two gamma distributions, a positive one with an early peak to capture the stimulus-induced dilation, and a slower negative one to generate a post-stimulus undershoot [15,240]. Because NO is a vasodilator and increases in GC activation are accompanied with increases in vessel diameter, we modeled the response of the vessel to NO using a single 6 second long gamma function matched only to the positive component of the HRFs observed *in vivo* [156]:

$$h(t)=A\left(\frac{t^{\alpha_{1}-1}\beta_{1}^{\alpha_{1}}e^{-\beta_{1}t}}{\Gamma (\alpha_{1})}\right)$$ Eq 8

Where α_1_ = 4.5, β_1_ = 2.5, t is time and A is the amplitude (with units sec^-1^) which was normalized such that $\int_{0}^{\infty }dt\;h(t)=1$. Note that *h*(*t*) is unitless. The predicted diameter was calculated in Matlab and transmitted to COMSOL with Matlab Livelink to dynamically adjust vessel diameter (**S6 Fig**):

$$\Delta diameter(t)=m\int_{\tau =0}^{\infty }d\tau (h(\tau ){GC}_{f}(\left[NO(t-\tau )\right])-\varphi )$$ Eq 9

The fractional change in diameter of the arteriole was the deviation of the convolution of the HRF (**Eq 8**) and past fractional GC activity (**Eq 7**) from its initial state (φ = GC_f_([EC_50_])) multiplied by the sensitivity of the arteriole to NO (m which has units of $\frac{\Delta diameter,\;\%}{\Delta GC\;activation,\;\%}$). This convolution was performed at each time step so that COMSOL could recalculate Fick’s diffusion equation given the new vascular diameter. Because a larger arteriole will supply more hemoglobin which scavenges more NO, this creates a dynamic model in which vasodilation was linked to changing NO degradation via a changing vessel diameter. The time-dependent changes in parenchymal NO production, R_x_(t), was kept constant for the initial 6 seconds of the simulation to both allow equilibration at steady state (*D*_*NO*_∇^2^[*NO*]+*R*_*x*_(*t*) = 0) and to provide enough previously experienced NO in the smooth muscle to convolve with the kernel, h(t). After 6 seconds the arteriole was allowed to adjust its diameter in response to changing NO concentrations, but only data acquired 15 seconds into the simulations was used to eliminate the influence of hemodynamics associated with the process of reaching steady state.

### Evaluating hemodynamics resulting solely from the HRF and arteriole sensitivity, m

The hemodynamic response imposed in the model is the result of a convolution of a kernel, h(t), whose magnitude is modified by the arteriole sensitivity to NO, m $\frac{\Delta diameter,\;\%}{\Delta GC\;activation,\;\%}$ (**Eq 9**). The shape of the chosen kernel has some bearing on hemodynamics. To isolate any dynamics that were a direct result of the chosen kernel and “m”, h(t) was convolved at integer values of m (m = 1,2,3,4 or 5) with a step function of increased GC activity (1%), a one second pulse of increased GC activity, and a white noise variation in GC (30 Hz, low pass filtered < 2 Hz) to emulate the analogous inputs of the diffusion-deformation model. NO diffusion and arteriole deformation were not a component of this simulation, as the goal was to isolate the hemodynamic of the kernel and “m” alone. The hemodynamic response to imposed changes in GC activity was evaluated using a modified version of Eq 9.

$$\Delta diameter(t)=m\int_{\tau =0}^{\infty }d\tau (h(\tau )GC(t-\tau ))$$ Eq 10

Where h(t) was the kernel, GC was the imposed changes in GC activity, “m” was the arteriole sensitivity to NO, t was the time, and *τ* was a dummy variable used for integration. The resulting HRF and power spectrum of the hemodynamic response was evaluated using identical methods to the simulations coupled to COMSOL.

### Parenchyma

NO is both produced and degraded in the parenchyma, although the rate of NO degradation within this region is much lower than the degradation rate of NO in the lumen. NO diffusion into the parenchyma was modeled with a reflecting (no flux) boundary condition at the radial boundaries of the simulated domain. Parenchymal NO production was geometrically varied between three models: uniform, regional, and proximal. In the uniform model, NO production was produced equally within the parenchymal domain. In the regional model, NO production within 50 μm was set to be 3.8 fold greater than the tissue further than 50 μm to mimic the increased perivascular density of nNOS neurons close to the vessel [33]. In the proximal model, all NO production in the parenchyma was restricted to within 2 μm of the arteriole wall. NO degradation in the parenchyma was dependent on the NO, oxygen, and cell concentration and expressed using the following equation [77]:

$$R_{parenchyma}={-k}_{O_{2}}[O_{2}][NO][Cell]$$ Eq 11

Where k_O2_ = 5.38 • 10^−4^ M^-1^s^-1^(cell/ml)^-1^ (**Table 1**) and the density of cellular sinks in the tissue ([Cell]) was chosen to be 10^8^ cell/ml, as was previously used for NO diffusion modeling in parenchymal tissue [235]. Note that the degradation rate of NO in the parenchyma was not uniform throughout the tissue because the oxygen content of the parenchyma changes with distance from the arteriole. Because the rate of NO degradation is proportional to the oxygen content of the tissue (**Eq 11**), the oxygen rich region of the parenchyma near the arteriole will have a higher degradation rate of NO than distant from the arteriole where the oxygen concentrations fall off. For all of the simulations presented with the exception of those in Fig 7, the oxygen concentrations changed slowly enough in time that they could be assumed to be at steady state. This allowed us to use the Krogh model, as it gave oxygen profiles identical to full simulations of diffusion of oxygen using Fick’s equations with little computational overhead. However, for simulations where rapid and large manipulations of oxygen levels were performed (**Fig 7**), we simulated the diffusion and consumption of oxygen in the parenchyma (**Eq 3**). Within the NO producing region of the parenchyma, NO production/degradation was accounted for with a production rate P_NO_(t) so that, overall:

$$R_{parenchyma}(t)=P_{NO}(t)-k_{O_{2}}[O_{2}][NO][Cell]$$ Eq 12

For steady-state simulations, P_NO_(t) was a constant production rate that was parametrically varied. For time-dependent simulations, P_NO_(t) was modified to be a pulse of increased NO production or white noise with a mean of the EC_50_ for guanylyl cyclase activity (8.9 nM).

The impact of parenchyma capillaries on NO degradation was modeled in S4 and S5 Figs. Capillary consumption of NO was estimated as the sum of NO scavenging in plasma and in the RBC. For these simulations, Eq 11 was modified to include the average hemoglobin supplied by the microcirculation. Because capillary diameter is similar to the size of RBCs, no cell free layer exists, so the equations for the degradation rate of NO in capillaries is similar to that in the RBC core (Eq 4) but with capillary hematocrit values (Hct_cap_, 0.23) [139]. The degradation rate in the capillaries is given by:

$$R_{capillary}(t)={-k}_{RBC\;Hb}{Hct}_{cap}[NO][{Hb}_{RBC}]-k_{CFL\;Hb}(1-{Hct}_{cap})[NO][{Hb}_{CFL}]$$ Eq 13

Since capillaries only compose 1% of the parenchymal volume [140], the scavenging of NO by capillaries in the parenchyma was obtained by multiplying R_capillary_ by the volume fraction of the capillaries in the parenchyma, f_c_ [140]. The final equation for parenchyma NO reactions when including parenchymal capillaries (R_pc_) was thus:

$$R_{pc}(t)=P_{NO}(t)-k_{O_{2}}[O_{2}][NO][Cell]-f_{c}R_{capillary}$$ Eq 14

### Power Spectrum Calculations

We investigated the preferred frequency of vasodynamics in the model by introducing a white Gaussian noise production rate of NO (30 Hz, low pass filtered < 2 Hz, 25 minute duration) in the parenchyma within 2 μm of the arteriole wall (proximal model). NO production was initially set such that GC activity in the smooth muscle was at EC_50_ (8.9 nM) and the variance from a white Gaussian noise change in NO production was chosen such that there was no change in vessel diameter exceeding ±5%. Vessel sensitivity was set to $m=4\;(\frac{\Delta diameter,\;\%}{\Delta GC\;activation,\;\%})$ unless otherwise indicated. The power spectral density was calculated from the arteriole response in the model using the Chronux toolbox version 2.11 (http://chronux.org, function: mtspectrumc). We used 101 averages for a frequency resolution of 0.067 Hz.

### Calculation of the hemodynamic response function

The relationship between neural activity and vessel dynamics is often considered a linear time-invariant (LTI) system [239,241,242] which allows for the effective hemodynamic response function to be calculated numerically using the relationship

$$H_{(k+1)\times 1}={\left(T^{T}T\right)}^{-1}T^{T}V_{(q+k)\times 1}$$ Eq 15

Where H is the effective HRF, V is the vascular response, and T is a Toeplitz matrix of size (q+k) × (k+1), containing measurements of normalized neural activity (n) [156].

$$T(\overset{⃑}{n})=\left(\begin{array}{cccccc} 1 & n_{1} & 0 & 0 & \cdots & 0\\ 1 & n_{2} & n_{1} & 0 & \cdots & 0\\ \vdots & \vdots & n_{2} & n_{1} & \cdots & \vdots \\ \vdots & n_{k} & \vdots & n_{2} & \cdots & n_{1}\\ \vdots & 0 & n_{k} & \vdots & \cdots & n_{2}\\ \vdots & \vdots & \vdots & n_{k} & \ddots & \vdots \\ 1 & 0 & 0 & 0 & \cdots & n_{k} \end{array}\right)$$ Eq 16

Note that this method makes no assumptions about the shape of the effective HRF. To evaluate the effective HRF produced in the model we performed the same calculation using a NO production rate (n) in place of neural activity, where n was white Gaussian noise.

### Estimating perivascular mitochondrial inhibition

Although NO dilates arteries, resulting in increased blood flow and O_2_ delivery to the tissue, it can also compete with O_2_ at the mitochondrial cytochrome c oxidase (CcO) to inhibit aerobic respiration and facilitate the generation of free radicals [138,227]. Under physiologic conditions, inhibition of CcO by NO is minimal and reversible [57,137,243,244], but under conditions of high NO and/or low O_2_, CcO can be permanently inhibited [148]. Permanent inhibition of CcO occurs at nominal NO and O_2_ concentrations of 1000 nM and 130 μM, respectively [148] which is equivalent to 12.5% CcO activity using a competitive model of inhibition:

$$V_{O_{2}}=\frac{\left[O_{2}\right]}{\left[O_{2}]+\zeta_{O_{2}}(1+\frac{\left[NO\right]}{\zeta_{NO}}\right)}$$ Eq 17

Here, V_O2_ is the fractional activity of CcO, ζ_O2_ = 210, ζ_NO_ = 0.225, and [O_2_] and [NO] are the respective oxygen and NO concentrations, expressed in nM [56,137,138]. Because permanent inhibition of CcO is likely pathological (V_O2_ ≤ 12.5%), it is unlikely that physiological NO-mediated NVC produces this combination of NO and O_2_ concentrations. The extent of CcO inhibition in the proximal, regional, and uniform models was evaluated as the transverse cross-sectional area (200 μm from the axial boundary) of the parenchyma with V_O2_ ≤ 12.5% divided by the area of the total parenchyma, excluding the vessel domains (lumen, endothelium and smooth muscle).

### Simulating diffusion in a deforming domain

The convection-diffusion equation in the deforming domain is solved using the arbitrary Lagrangian-Eulerian (ALE) method. The displacements in in the RBC core and the cell free layer during vasodilation and constriction were modeled with a displacement field, ***u***_*m*_, usually referred to as the mesh displacement [245]. Since the displacements are all only in the radial direction, the displacement field was modelled by a linear model (Eq 18), which ensures that the radial displacement (*u*_*mr*_) at the center of the vessel is zero and is equal to the vasodilation (*u*_*mr*_ = *u*_*vessel*_) at the vessel wall (r = *R*_*vessel*_). The axial component of the mesh displacement (*u*_*mz*_) is always zero.

$$u_{mr}=(r/R_{vessel})u_{vessel}$$ Eq 18

We use the displacement to calculate the deformation gradient (***F***_*m*_) and the Jacobian determinant (*J*_*m*_), which can be used to transform spatial gradients and integrals:

$$F_{m}=I+\nabla u_{m};\;J_{m}=Det[F_{m}]$$ Eq 19

Where ***I*** in Eq 19 is the identity tensor.

The deformation gradient and the Jacobian determinant are used to transform all the gradients and integrals from the physical coordinates (***X***_*p*_) to the mesh coordinates (***X***_*m*_).

For a scalar field, *c*:

$$\nabla_{X_{P}}c={F_{m}}^{-T}\nabla_{X_{m}}c$$ Eq 20

For a vector field, ***u***:

$$\nabla_{X_{P}}u=\nabla_{X_{m}}u\;{F_{m}}^{-1}$$ Eq 21

$$\nabla_{X_{P}}.u=\frac{1}{J_{m}}\nabla_{X_{m}}.(J_{m}\;{F_{m}}^{-1}u)$$ Eq 22

An infinitesimal volume in the deformed physical coordinates is scaled by the Jacobian determinant.

$$dV_{X_{p}}=J_{m}dV_{X_{m}}$$ Eq 23

The advection-diffusion equation is written in the physical coordinates is given by Eq 24. In the mesh coordinates, the equation takes its ALE form:

$$\frac{dc}{dt}=\frac{\partial c}{\partial t}+v_{f}.\nabla_{X_{p}}c=D_{NO}\nabla_{X_{p}}.(\nabla_{X_{p}}c)+R_{RBC\;core/CFL}$$ Eq 24

$$J_{m}(\frac{\partial c}{\partial t}+{(v}_{f}-\frac{\partial u_{m}}{\partial t}).{{F_{m}}^{-T}\nabla }_{X_{m}}c)=D_{NO}\nabla_{X_{m}}.(J_{m}{{F_{m}}^{-1}{F_{m}}^{-T}\nabla }_{X_{m}}c)+J_{m}R_{RBC\;core/CFL}$$ Eq 25

Where ***v***_*f*_ is the velocity of the fluid. The displacement (***u***_*s*_) in the tissue (smooth muscle and brain tissue) is modeled by linear elasticity with a Poisson’s ratio (υ) of 0.45. The displacement of the tissue is governed by equation Eq 26, where μ and λ are the shear modulus and Lamé’s first parameter, respectively.

$$\nabla .\sigma_{s}=0,where\;\sigma_{s}=\lambda (Tr[\epsilon_{s}])I+2\mu \epsilon_{s}$$ Eq 26

$$\epsilon_{s}=\frac{1}{2}(F_{s}+{F_{s}}^{T})$$ Eq 27

The linearized Lagrange strain is given by ***ϵ***_*s*_.The displacement gradient for tissue placement, ***F***_***s***_, is defined in a manner similar to Eq 19.

The diffusion equation in the tissue is given in its physical coordinates (***X***_*p*_) by

$$\frac{dc}{dt}=D_{NO}\nabla_{X_{p}}.(\nabla_{X_{p}}c)+\frac{1}{J_{s}}R_{Smooth\;Muscle/Tissue}$$ Eq 28

As the volume of the tissue changes (as given by Eq 23, with *J*_*s*_ replacing *J*_*m*_), the NO production rate per unit mass of the tissue is kept the same, whereas we adjust the NO production rate per unit volume by a factor of 1/*J*_*s*_ to account for any volume changes. The diffusion equation in Lagrangian (tissue) coordinates (***X***_*s*_) is given by:

$$J_{s}\left(\frac{dc}{dt}\right)=D_{NO}\nabla_{X_{s}}.(J_{s}{{F_{s}}^{-1}{F_{s}}^{-T}\nabla }_{X_{s}}c)+R_{Smooth\;Muscle/Tissue}$$ Eq 29

## Supporting information

S1 Fig Convection has a negligible effect on perivascular NO concentrations for both Poiseuille and blunted flow. In this model, the flow of blood is driven by a pressure gradient along the axis of the vessel. The concentration of NO in the blood entering the vessel is 0. For Poiseuille flow, the viscosity of the fluid was set at 1.2 cP. To generate blunted flow, the viscosity of the red blood cell core was set to 7cP [141,142]. To generate different centerline velocities, the axal pressure gradient was varied. The vessel diameter is fixed at 20μm. Quantification of NO levels were made at the center of the vessel, 200 μm from either end. Simulations were performed using the dynamic proximal model with a NO production rate of 0.02 M/s corresponding to 50% GC activity with no flow. A) Poiseuille velocity profile. B) Plot NO concentration and GC activation as a function of centerline velocity for the simulated arteriole. The physiologically plausible range of velocities is denoted the pink region. Within physiologic blood flow velocities in a 20 μm diameter arteriole [246,247], transport causes a small change in [NO] (0.1% change in GC activation). The pink diamond indicates a physiologic flow in a 20 μm diameter arteriole, while the green diamond is approximately tenfold higher, comparable to the centerline velocity in a 200 μm diameter arteriole [247]. C) Radial [NO] profiles 200 μm at physiologic (pink) and extreme (green) blood flow velocities. Pink and green data diamonds in B are plotted as perivascular [NO] profiles in C. D) Blunted velocity profile. E) Plot NO concentration and GC activation as a function of centerline velocity for the blunted profile. F) Radial [NO] profiles 200 μm the at physiologic (pink) and extreme (green) blood flow velocities for the blunted profile.
(TIF)

S2 Fig Axial and radial gradients of NO. In this model, the flow of blood is driven by a pressure gradient along the axis of the vessel. The flow has a blunted profile and the vessel has a diameter of 20μm. The centerline velocity is 12 mm/s. Simulations were performed using the dynamic proximal model with a NO production rate in the parenchyma of 0.02 M/s corresponding to 50% GC activity with no flow. The concentration of NO in the blood entering the vessel at the top is 0. A) Plot of NO concentration in the radial and axial directions. Color denotes NO concentration. The black vertical line 200 μm from the top is where perivascular NO concentrations were evaluated for other simulations. B) Enlargement of the area where blood (with 0 NO) enters. In the first few microns, the concentration of NO in and near the vessel lumen is lower and but equilibrates within ~25 μm. Black, orange, and green bars indicate the location of the lumen, smooth muscle and parenchyma respectively C) Schematic of the velocity profile.
(TIF)

S3 FigChange in smooth muscle NO from a dynamic increase in flow.Simulations were performed using a static proximal model with a NO production rate in the parenchyma of 0.02 M/s corresponding to 50% GC activity with no flow. A) Schematic of the blunted velocity profile. B) Plot of the centerline velocity profile in time. The centerline velocity of blood flow through a 20 μm diameter arteriole was increased by 50% from 10 mm/s to 15 mm/s by increasing the pressure difference between the inlet and outlet of the lumen. C) Plot of the NO concentration in the smooth muscle at the midpoint of the vessel (200μm from the end) versus time. The increase in velocity resulted in a decrease in GC activity by ~0.03%. which would correspond to a 0.1% constriction for an arteriole with sensitivity of m = 3 $\frac{\Delta diameter,\;\%}{\Delta GC\;activation,\;\%}$.(TIF)

S4 Fig Impact of the location of NO production on NO concentration in the smooth muscle and tissue with capillaries. All simulations in this figure used a static model. The geometry of the NO production and the vessel diameter were varied. The oxygen concentration was calculated from the Krogh model. This model includes increased NO scavenging by the blood in the capillaries (Eq 13). A) Schematic showing the three simulated distributions of neuronal NO production relative to the vasculature. In the uniform model, neuronal NO-production is uniformly distributed through the parenchyma. In the regional model, there is a higher density of neuronal NO production near the vessel (within 50 μm) [33]. In the proximal model, all neuronal NO is produced within 2 micrometers of the arterial wall [33,123,248]. B) Plot of NO concentrations versus radial distance for each of the three models where the production rates have been chosen to yield equal concentration of NO in the smooth muscle layer (NO production rate for proximal: 0.025 M/s; regional: 0.141 M/s; uniform: 0.2 M/s). Note that the concentration of NO in the parenchyma is very different for each of these models. C) Relationship between [NO] in the smooth muscle and percent of maximal guanylyl cyclase activity in the model, based on experimental data in [51,118,249]. D) Plot showing percent of maximal guanylyl cyclase activation in the smooth muscle as a function of the NO production rate and vessel diameter in each of the three geometries. Superimposed curves show 10, 50, and 90% of maximal guanylyl cyclase activation. White boxes show the NO production rates and vessel diameters shown in B.
(TIF)

S5 Fig Extent of the NO inhibition of mitochondrial respiration depends on the location of NO production with capillaries. All simulations in this figure used a static model. The geometry of the NO production and the vessel diameter were varied. The oxygen concentration was calculated from the Krogh model. This model includes increased NO degradation by the blood in the capillaries (Eq 13). A) Plots of cytochrome c oxidase (CcO) activity as a function of radial distance for the uniform, regional, and proximal models. The vessel diameter was set to 40 micrometers, and NO production rates have been set so that there is 90% of maximal GC activation in the smooth muscle. Insets show oxygen and NO concentrations as a function of radial distance. Oxygen concentration curves are matched to *in vivo* measurements [130,250]. B) The fraction of the parenchyma where CcO activity is inhibited to <12.5% of normal as function of various NO production levels and vessel diameters for each of the three different NO production geometries. Red boxes indicate simulations plotted in (A). Note that for the regional and uniform NO production geometries, CcO inhibition becomes an issue at a wider range of NO production levels. For the proximal production case, inhibition of respiration by NO only occurs at the highest levels of NO production.
(TIF)

S6 Fig Schematic showing how diffusion and deformation simulations were coupled. A) NO gradients surrounding an arteriole is evaluated using Fick’s equation. B) The recent history of NO levels in the smooth muscle domain is converted to GC activation and convolved with a kernel to account for the signaling cascade that converts GC activation into dilation. This kernel was chosen to match the temporal dynamics of neurally-evoked dilation. C) Arteriole diameter is adjusted depending on the output of the kernel with increases or decreases in NO driving to dilation and constriction respectively. Diffusion of NO is then calculated and used re-evaluated using the new arteriole geometry. Adjustments to arteriole diameter using this cycle are made at each time step.
(TIF)

S7 Fig Matching the model dynamics to dilations observed *in vivo*. The diameter change mouse pial arteriole diameters in the somatosensory cortex in response to a single and 10-second-long puff to the whiskers [1] (black) were compared to the model with a variable RBC core (red) or constant RBC core (blue). The response kernel of the vessel was fitted to the positive component of the hemodynamic response function (HRF) from a single whisker stimulation (inset) and the slope of vessel sensitivity to NO, m, was set to 3. Allowing the degradation of NO to dynamically change with arteriole diameter imposed a post-stimulus undershoot that was not present when the RBC core diameter was held constant.
(TIF)

S8 FigEffective HRF of dynamic models is a linear system.All simulations in this figure used a dynamic model. A) The variable and constant RBC core model effective HRF shown is deconvolved from 12 minutes of white noise NO production when m = 4 $\frac{\Delta diameter,\;\%}{\Delta GC\;activation,\;\%}$. B) Convolution of the NO production rate with the kernel has a high predictive value for estimating the arteriole response for a different 12 minutes of data. R^2^ values shown are from m = 1,2,3,4, and 5. C) Example 300 seconds of data showing the difference between the response in the model and an approximation using the kernels shown in A. Example shown for m = 4.(TIF)

S9 Fig The undershoot and vasomotion cannot be generated by the chosen hemodynamic response function alone and the m parameter linearly scales its amplitude. This model is solely a convolution of the kernel, h(t), with an imposed ΔGC activity and does not contain any COMSOL components that account for the diffusion of NO or geomsetry changes from arteriole dilation. A) A 1% increase in ΔGC activity (black) was imposed for 20 seconds or 1 second and convolved with the kernel, h(t), as shown in Eq 10 for varying degrees of arteriole sensitivity (m = 1,2,3,4 & 5). Although a larger “m” increased the magnitude of the expected vascular response (red), convolution of GC activity with the kernel was not able to generate the post-stimulus undershoot. B) Convolution of the kernel, h(t), with a white noise ΔGC activity (black) produced a predicted hemodynamic response (red). Only 60 seconds of the 50-minute simulation is shown. C) The HRF and power spectrum of the vascular response in B do not produce a post-stimulus undershoot or vasomotion-like oscillations, indicating that they are not emergent phenomena due to the chosen shape of h(t) or value of “m”.
(TIF)

S10 FigPost-stimulus undershoot and vasomotion with different HRFs.All simulations in this figure used a dynamic model. An arteriole sensitivity of m = 4 $\frac{\Delta diameter,\;\%}{\Delta GC\;activation,\;\%}$ was used for these simulations. A) Plots of the HRFs used in these simulations. The HRFs used have faster (peak at 0.5 seconds, cyan) or slower (2.1 seconds, black) dynamics than the kernel used in all other simulations (1.5 seconds, red) Note that the integrated areas of all the HRFs are the same. B) Effective HRF evaluated from deconvolving the response to white noise NO stimulation. C) Power spectrum of the responses of each of the models to white noise. Slower hemodynamic responses to NO (black) amplified the post-stimulus undershoot and 0.1–0.3 Hz oscillations. The faster hemodynamic response (cyan) did not have an appreciable post-stimulus undershoot or resonant frequency near 0.1–0.3 Hz. Due to the rapid response to NO, the hemodynamics from the fast kernel contained power in the higher frequencies (C, cyan).(TIF)

S11 Fig Impact of hematocrit on vasodynamics. All simulations in this figure used a dynamic model. In this figure, the hematocrit was varied. A) Increasing hematocrit increases the degradation of NO in the RBC core and reduces the size of the cell free layer. B) Baseline GC activation and arteriole diameter are predicted to decrease with increasing hematocrit and increase with decreasing hematocrit. C) Perivascular NO increases with low hematocrit and decreases with elevated hematocrit. The location of the smooth muscle is indicated in orange. D) Changing hematocrit does not alter the hemodynamic response function (note near complete overlap of effective HRFs) or the frequency response of the vessels (E).
(TIF)

S12 FigPost-stimulus undershoot and vasomotion persist with different GC activation levels.All simulations in this figure used a dynamic model. An arteriole sensitivity of m = 4 $\frac{\Delta diameter,\;\%}{\Delta GC\;activation,\;\%}$ was used. A) The perivascular NO concentration was evaluated for a baseline NO concentration eliciting 25% activation of the smooth muscle (green) or 50% GC activity with an EC_50_ of 3.9 nM and Hill coefficient of 2.1 [149] (gray). The simulation with an EC_50_ of 8.9 nM (Hill coefficient, 0.8) and baseline GC activity of 50% is shown in black for reference. Black and gray perivascular NO profiles overlap because the NO production rate was set such that there was 50% GC activity in the smooth muscle for both cases. B) Effective HRFs obtained by deconvolution of the hemodynamic response to white noise NO production. C) Power spectrum of each of the three conditions in response to white noise NO production. Decreasing baseline GC activity reduced the magnitude of the post-stimulus undershoot and bandpass-like properties of the system (green). Decreasing the EC_50_ of the relationship between NO and GC activity and increasing the Hill coefficient to 2.1 amplified the post-stimulus undershoot and bandpass-like properties (gray).(TIF)

S13 FigPost-stimulus undershoot and vasomotion persist with different parenchymal production and degradation rates of NO.All simulations from this figure used a dynamic model. An arteriole sensitivity of m = 4 $\frac{\Delta diameter,\;\%}{\Delta GC\;activation,\;\%}$ was used. A) The perivascular NO concentration was evaluated with the presence of capillaries (red) or with increasing amounts of background NO production (gray and light gray). The rates of NO production by the parenchyma distal to the proximal 2 μm of the arteriole was adjusted to compose either 0% (black), 10% (gray) or 40% (light gray) of the total NO produced. As a larger fraction of NO is produced distally, perivascular NO concentrations increase (gray and light gray) while the presence of capillaries depletes perivascular NO (red). B & C) B and C were evaluated by deconvolution of the hemodynamic response with a white noise NO production rate or by reporting the frequency spectrum of the hemodynamics respectively. The presence of capillaries (red) or an increased rate of background NO production (gray and light gray) had minimal effects on the undershoot present in the effective HRF or bandpass-like properties of the system from the proximal simulation (black).(TIF)
