# Supplementary figures and images for: Spatial and temporal patterns of nitric oxide diffusion and degradation drive emergent cerebrovascular dynamics
Source: PLoS Comput Biol. 2020 Jul 27;16(7):e1008069. doi: 10.1371/journal.pcbi.1008069 (PMC7410342; doi:10.1371/journal.pcbi.1008069)

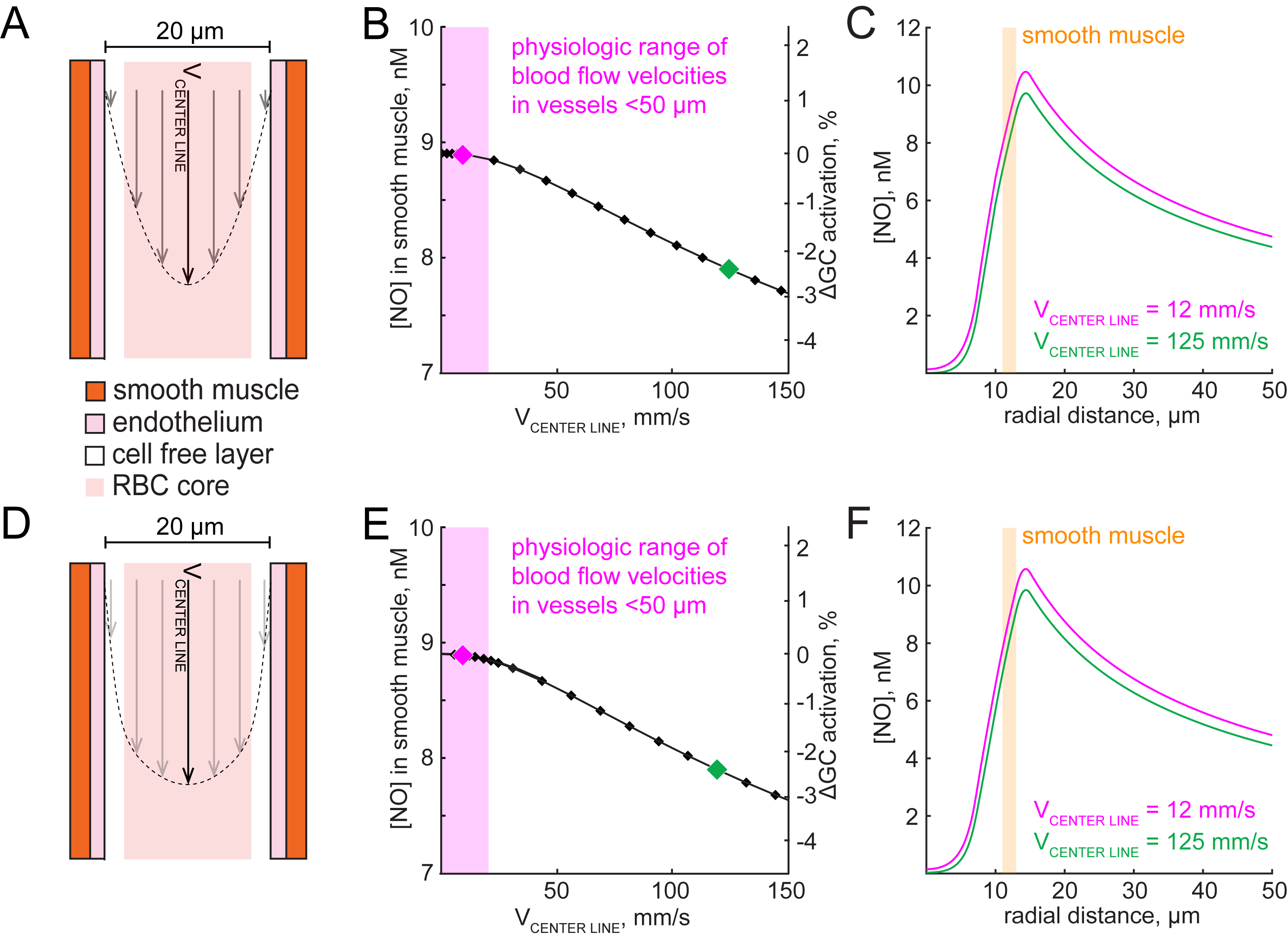

Supplement: S1 Fig — In this model, the flow of blood is driven by a pressure gradient along the axis of the vessel. The concentration of NO in the blood entering the vessel is 0. For Poiseuille flow, the viscosity of the fluid was set at 1.2 cP. To generate blunted flow, the viscosity of the red blood cell core was set to 7cP [141,142]. To generate different centerline velocities, the axal pressure gradient was varied. The vessel diameter is fixed at 20μm. Quantification of NO levels were made at the center of the vessel, 200 μm from either end. Simulations were performed using the dynamic proximal model with a NO production rate of 0.02 M/s corresponding to 50% GC activity with no flow. A) Poiseuille velocity profile. B) Plot NO concentration and GC activation as a function of centerline velocity for the simulated arteriole. The physiologically plausible range of velocities is denoted the pink region. Within physiologic blood flow velocities in a 20 μm diameter arteriole [246,247], transport causes a small change in [NO] (0.1% change in GC activation). The pink diamond indicates a physiologic flow in a 20 μm diameter arteriole, while the green diamond is approximately tenfold higher, comparable to the centerline velocity in a 200 μm diameter arteriole [247]. C) Radial [NO] profiles 200 μm at physiologic (pink) and extreme (green) blood flow velocities. Pink and green data diamonds in B are plotted as perivascular [NO] profiles in C. D) Blunted velocity profile. E) Plot NO concentration and GC activation as a function of centerline velocity for the blunted profile. F) Radial [NO] profiles 200 μm the at physiologic (pink) and extreme (green) blood flow velocities for the blunted profile. (TIF) [file pcbi.1008069.s001.tif]

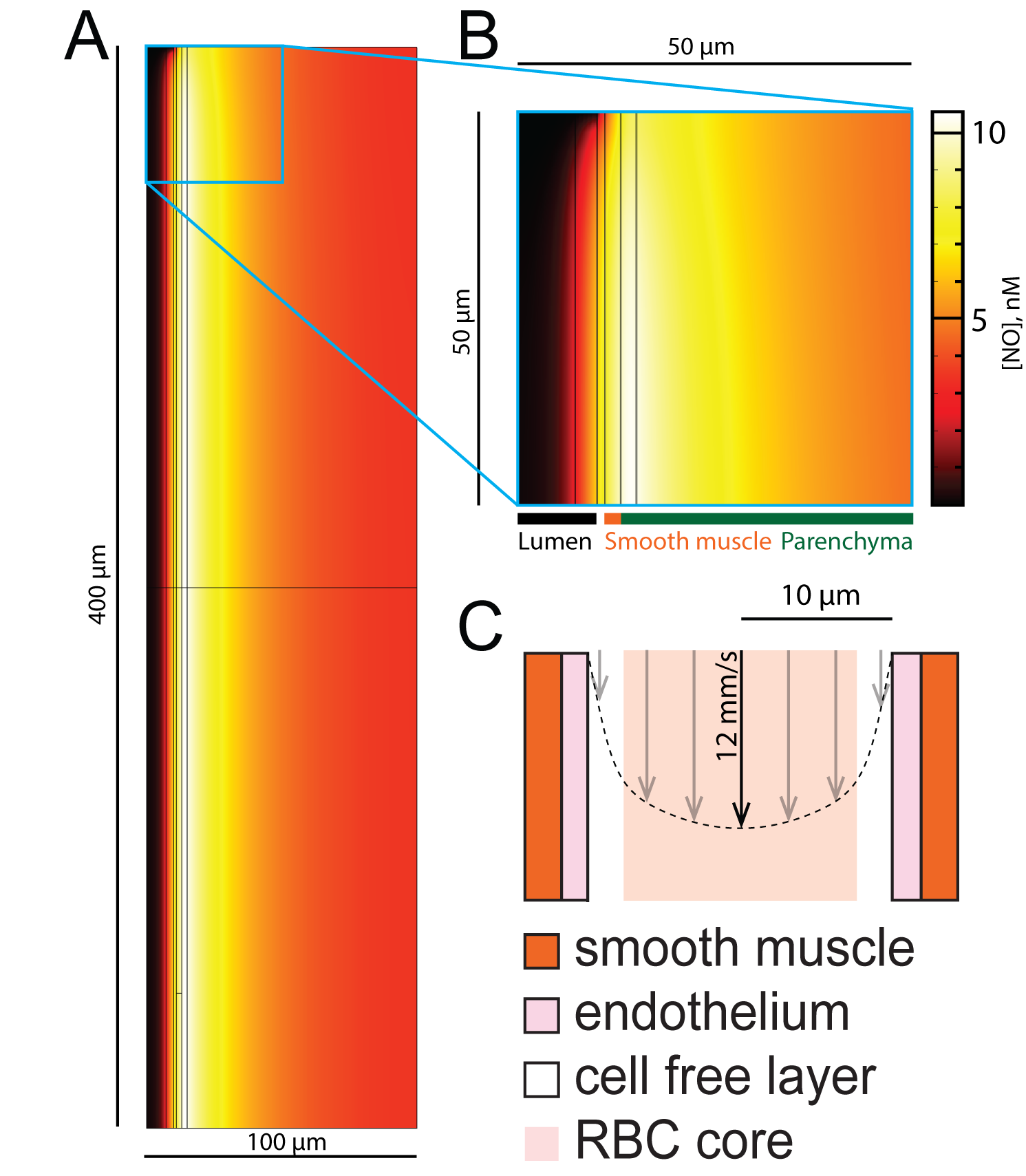

Supplement: S2 Fig — In this model, the flow of blood is driven by a pressure gradient along the axis of the vessel. The flow has a blunted profile and the vessel has a diameter of 20μm. The centerline velocity is 12 mm/s. Simulations were performed using the dynamic proximal model with a NO production rate in the parenchyma of 0.02 M/s corresponding to 50% GC activity with no flow. The concentration of NO in the blood entering the vessel at the top is 0. A) Plot of NO concentration in the radial and axial directions. Color denotes NO concentration. The black vertical line 200 μm from the top is where perivascular NO concentrations were evaluated for other simulations. B) Enlargement of the area where blood (with 0 NO) enters. In the first few microns, the concentration of NO in and near the vessel lumen is lower and but equilibrates within ~25 μm. Black, orange, and green bars indicate the location of the lumen, smooth muscle and parenchyma respectively C) Schematic of the velocity profile. (TIF) [file pcbi.1008069.s002.tif]

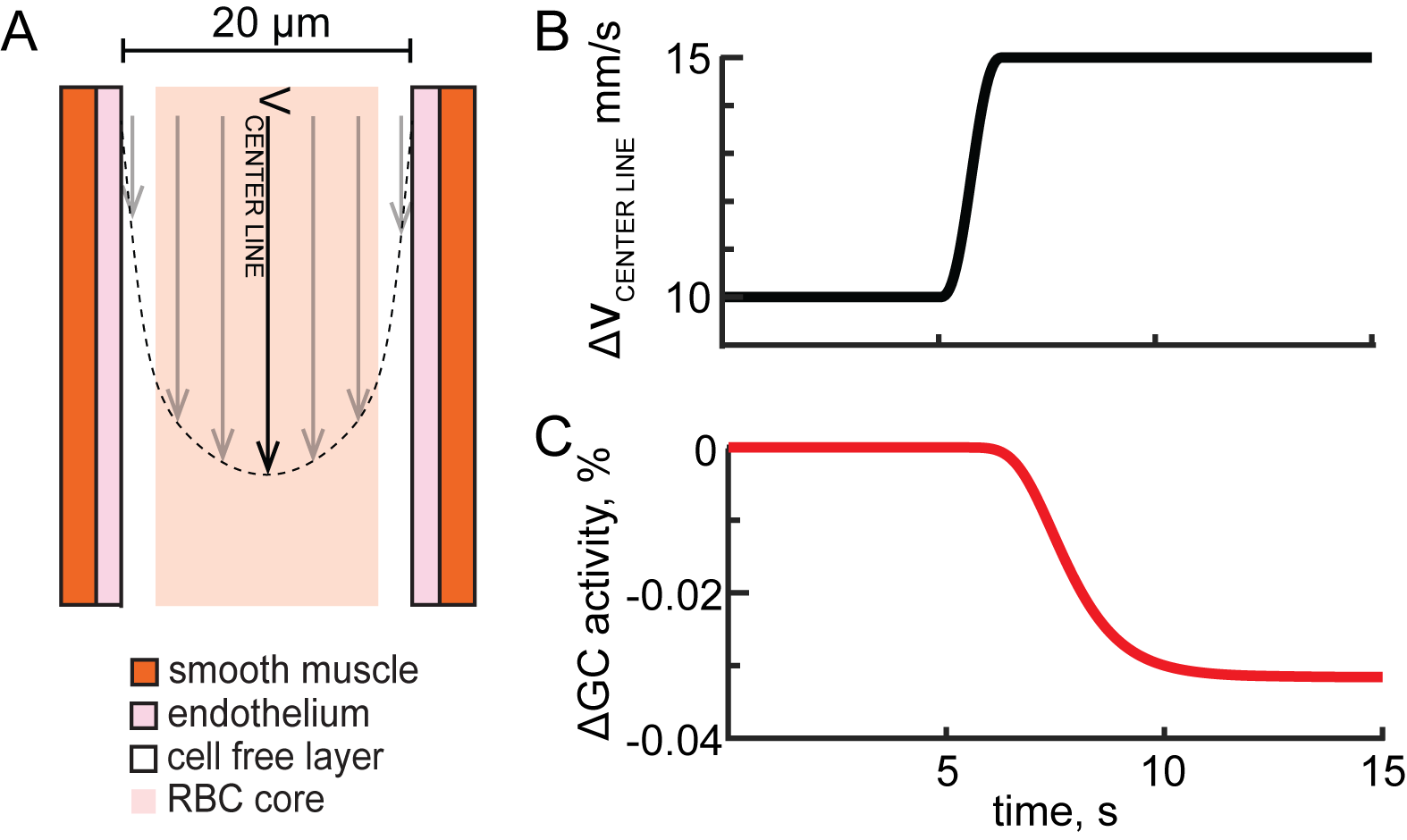

Supplement: S3 Fig — Simulations were performed using a static proximal model with a NO production rate in the parenchyma of 0.02 M/s corresponding to 50% GC activity with no flow. A) Schematic of the blunted velocity profile. B) Plot of the centerline velocity profile in time. The centerline velocity of blood flow through a 20 μm diameter arteriole was increased by 50% from 10 mm/s to 15 mm/s by increasing the pressure difference between the inlet and outlet of the lumen. C) Plot of the NO concentration in the smooth muscle at the midpoint of the vessel (200μm from the end) versus time. The increase in velocity resulted in a decrease in GC activity by ~0.03%. which would correspond to a 0.1% constriction for an arteriole with sensitivity of m = 3 Δdiameter,%ΔGCactivation,%. (TIF) [file pcbi.1008069.s003.tif]

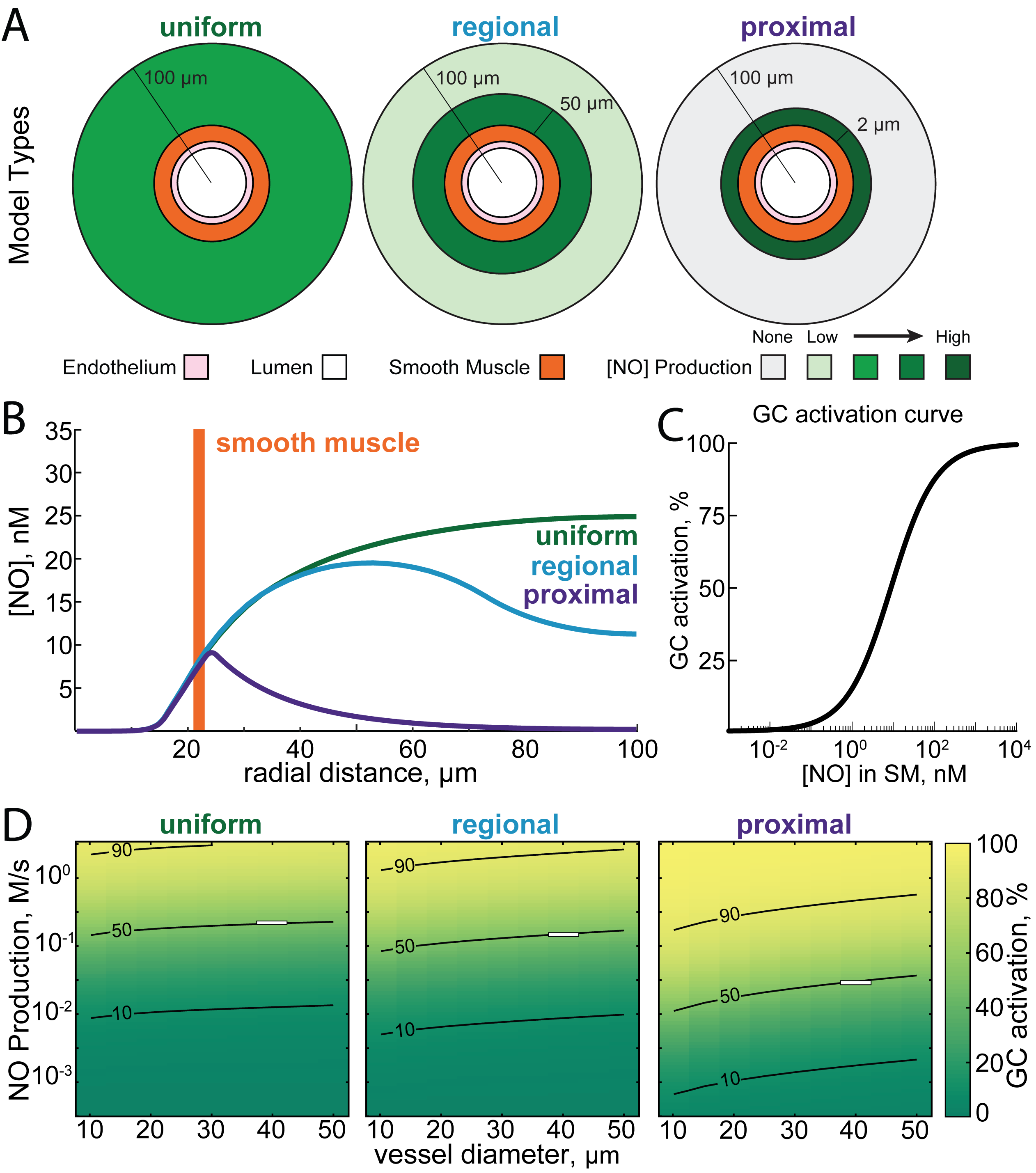

Supplement: S4 Fig — All simulations in this figure used a static model. The geometry of the NO production and the vessel diameter were varied. The oxygen concentration was calculated from the Krogh model. This model includes increased NO scavenging by the blood in the capillaries (Eq 13). A) Schematic showing the three simulated distributions of neuronal NO production relative to the vasculature. In the uniform model, neuronal NO-production is uniformly distributed through the parenchyma. In the regional model, there is a higher density of neuronal NO production near the vessel (within 50 μm) [33]. In the proximal model, all neuronal NO is produced within 2 micrometers of the arterial wall [33,123,248]. B) Plot of NO concentrations versus radial distance for each of the three models where the production rates have been chosen to yield equal concentration of NO in the smooth muscle layer (NO production rate for proximal: 0.025 M/s; regional: 0.141 M/s; uniform: 0.2 M/s). Note that the concentration of NO in the parenchyma is very different for each of these models. C) Relationship between [NO] in the smooth muscle and percent of maximal guanylyl cyclase activity in the model, based on experimental data in [51,118,249]. D) Plot showing percent of maximal guanylyl cyclase activation in the smooth muscle as a function of the NO production rate and vessel diameter in each of the three geometries. Superimposed curves show 10, 50, and 90% of maximal guanylyl cyclase activation. White boxes show the NO production rates and vessel diameters shown in B. (TIF) [file pcbi.1008069.s004.tif]

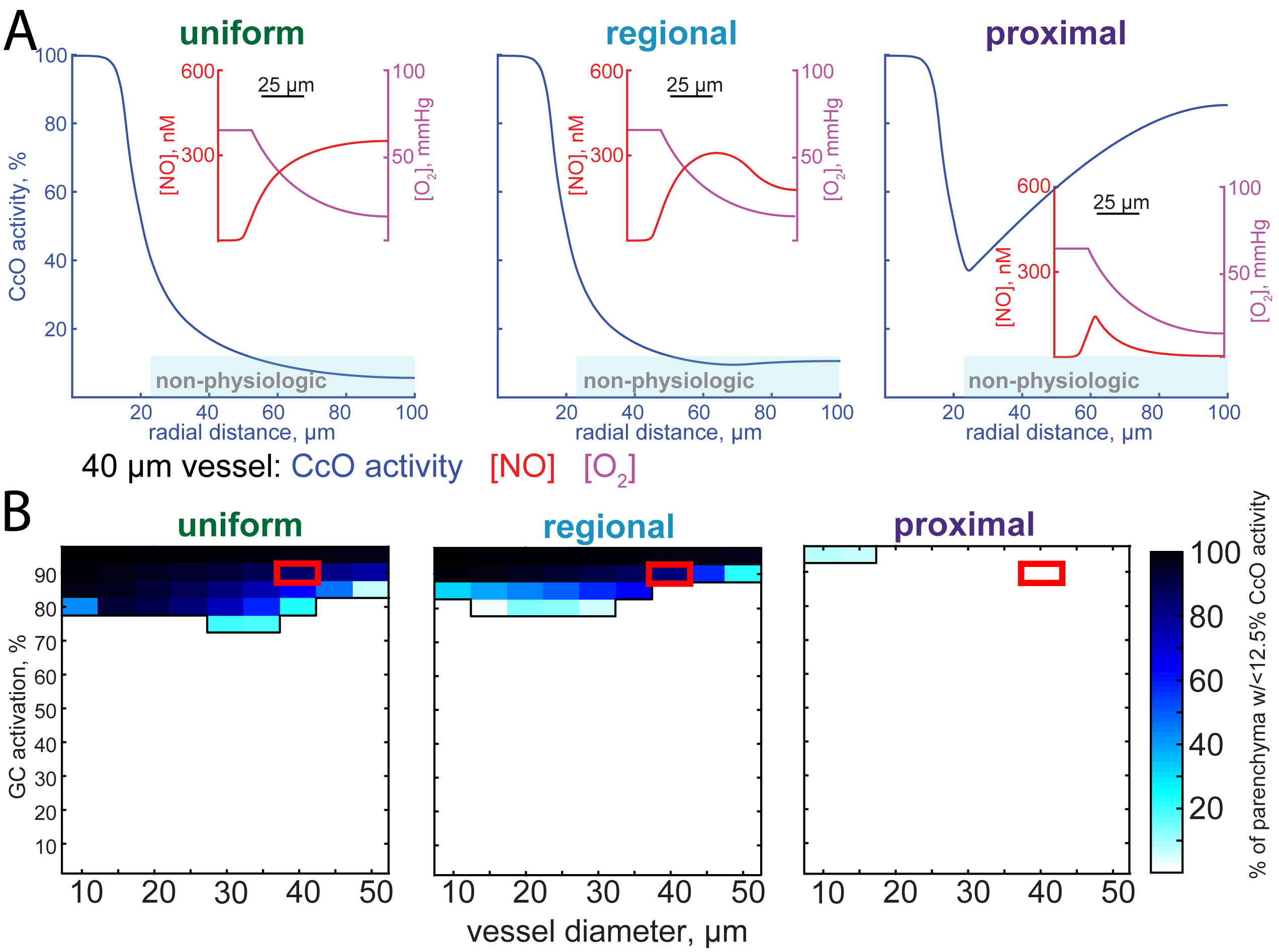

Supplement: S5 Fig — All simulations in this figure used a static model. The geometry of the NO production and the vessel diameter were varied. The oxygen concentration was calculated from the Krogh model. This model includes increased NO degradation by the blood in the capillaries (Eq 13). A) Plots of cytochrome c oxidase (CcO) activity as a function of radial distance for the uniform, regional, and proximal models. The vessel diameter was set to 40 micrometers, and NO production rates have been set so that there is 90% of maximal GC activation in the smooth muscle. Insets show oxygen and NO concentrations as a function of radial distance. Oxygen concentration curves are matched to in vivo measurements [130,250]. B) The fraction of the parenchyma where CcO activity is inhibited to <12.5% of normal as function of various NO production levels and vessel diameters for each of the three different NO production geometries. Red boxes indicate simulations plotted in (A). Note that for the regional and uniform NO production geometries, CcO inhibition becomes an issue at a wider range of NO production levels. For the proximal production case, inhibition of respiration by NO only occurs at the highest levels of NO production. (TIF) [file pcbi.1008069.s005.tif]

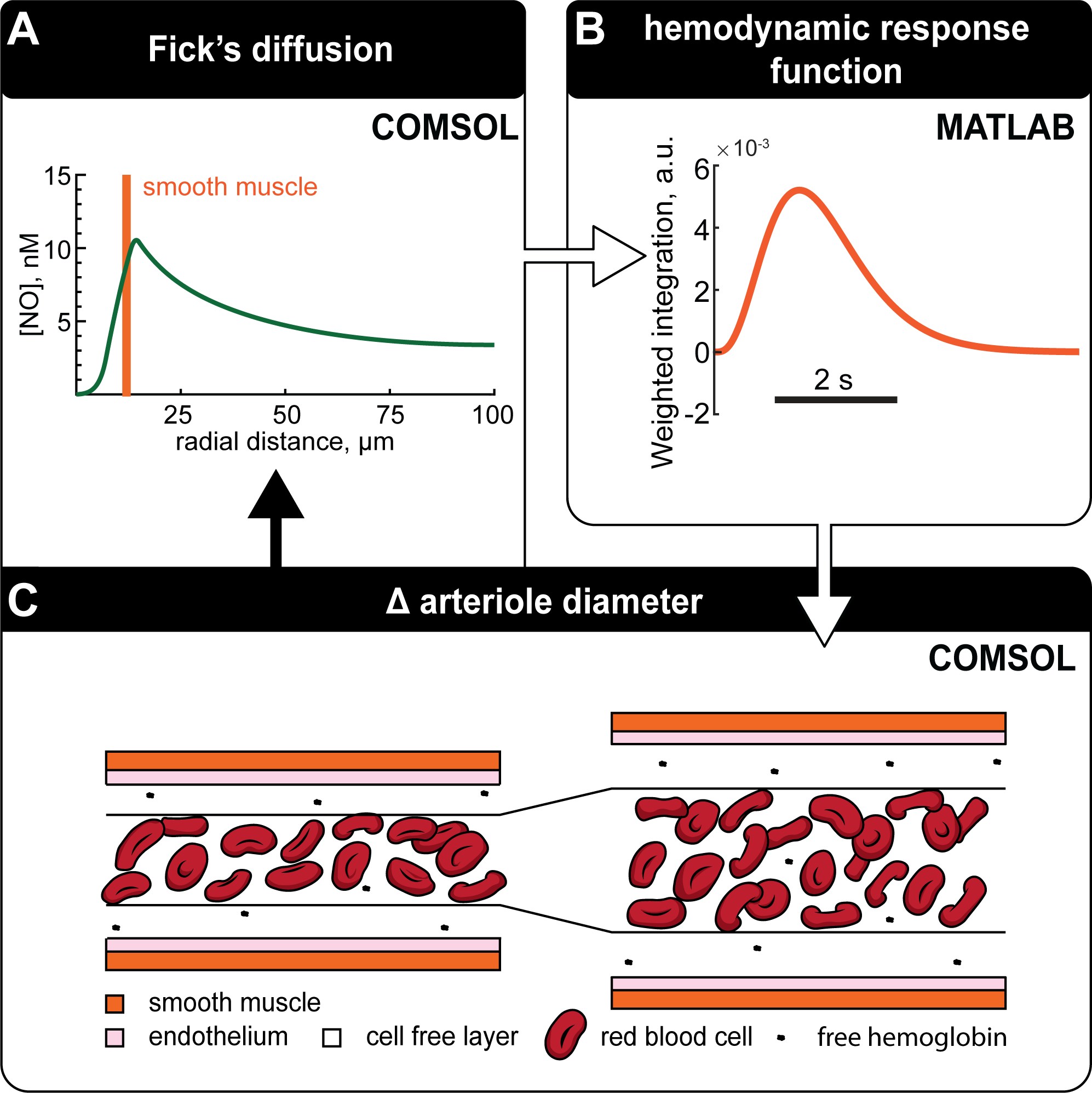

Supplement: S6 Fig — A) NO gradients surrounding an arteriole is evaluated using Fick’s equation. B) The recent history of NO levels in the smooth muscle domain is converted to GC activation and convolved with a kernel to account for the signaling cascade that converts GC activation into dilation. This kernel was chosen to match the temporal dynamics of neurally-evoked dilation. C) Arteriole diameter is adjusted depending on the output of the kernel with increases or decreases in NO driving to dilation and constriction respectively. Diffusion of NO is then calculated and used re-evaluated using the new arteriole geometry. Adjustments to arteriole diameter using this cycle are made at each time step. (TIF) [file pcbi.1008069.s006.tif]

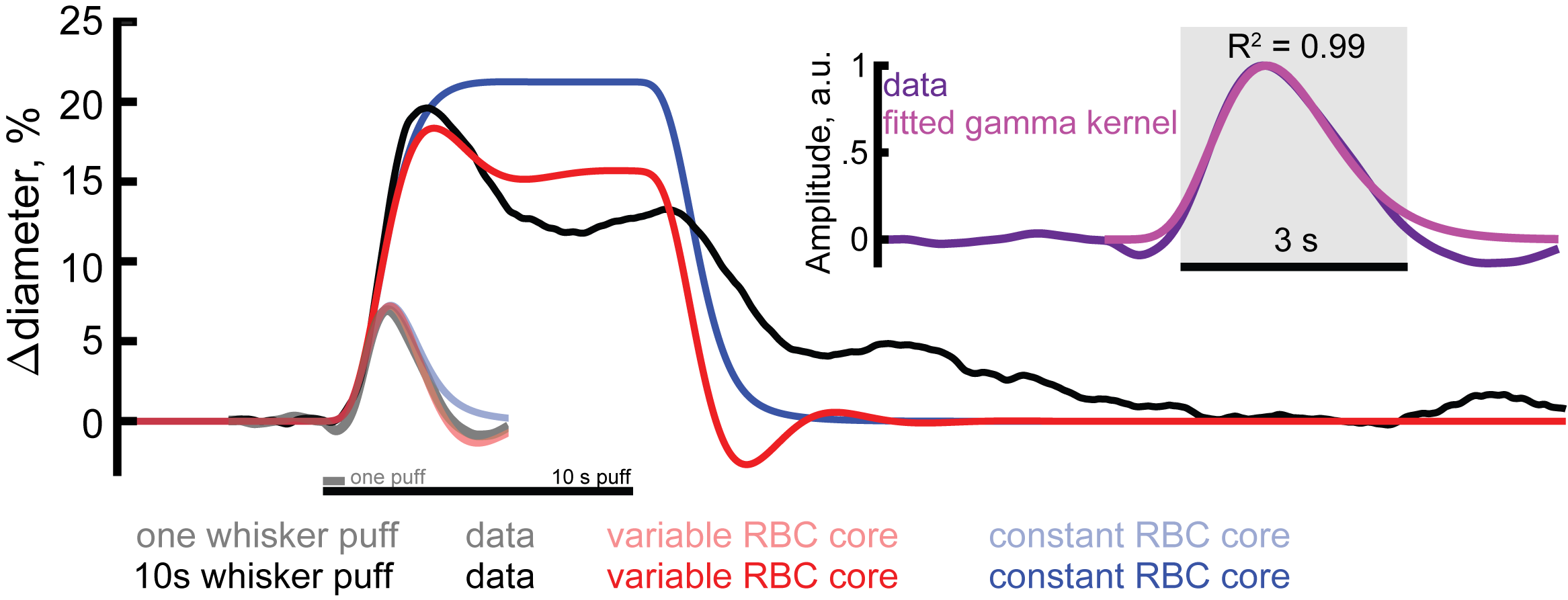

Supplement: S7 Fig — The diameter change mouse pial arteriole diameters in the somatosensory cortex in response to a single and 10-second-long puff to the whiskers [1] (black) were compared to the model with a variable RBC core (red) or constant RBC core (blue). The response kernel of the vessel was fitted to the positive component of the hemodynamic response function (HRF) from a single whisker stimulation (inset) and the slope of vessel sensitivity to NO, m, was set to 3. Allowing the degradation of NO to dynamically change with arteriole diameter imposed a post-stimulus undershoot that was not present when the RBC core diameter was held constant. (TIF) [file pcbi.1008069.s007.tif]

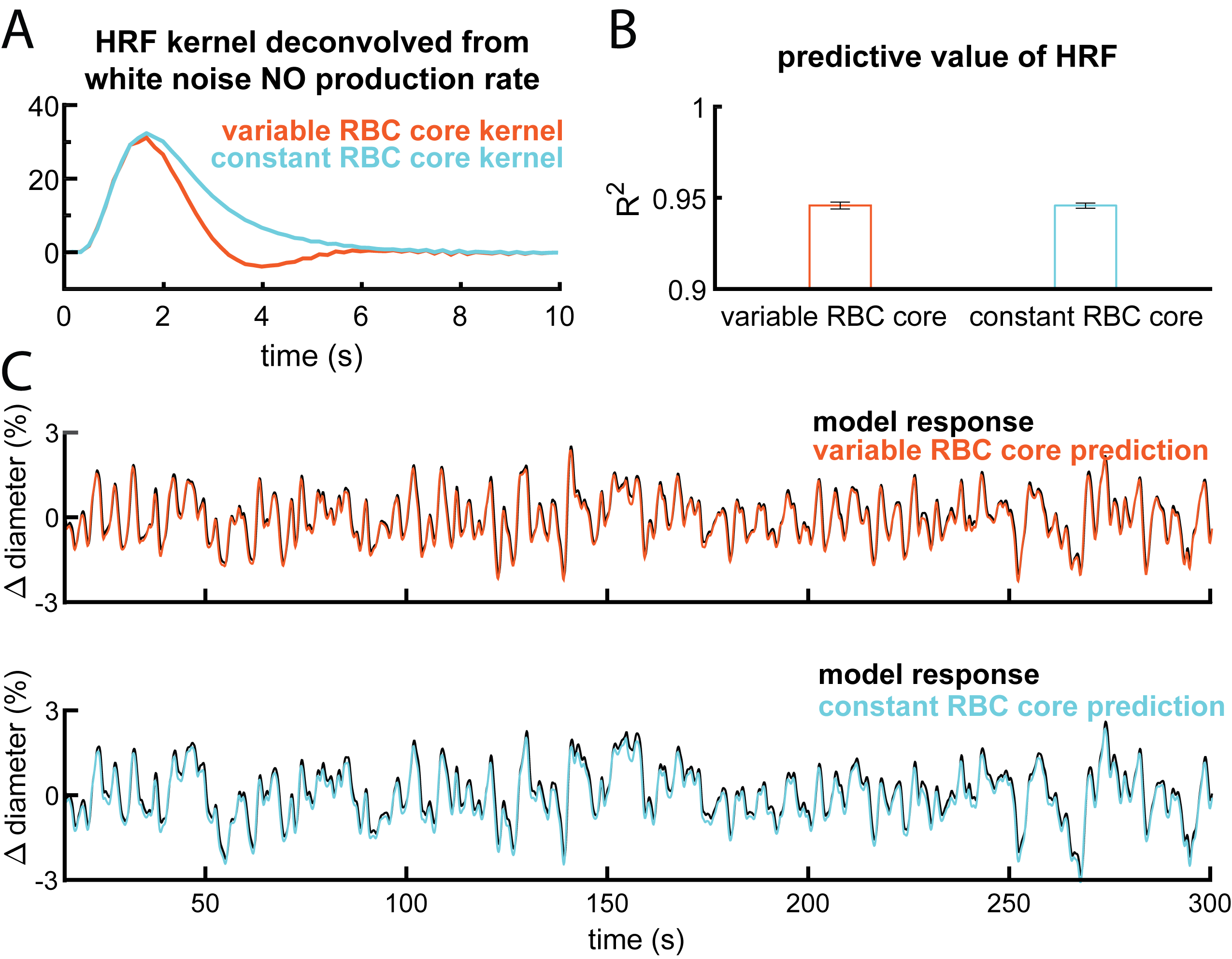

Supplement: S8 Fig — All simulations in this figure used a dynamic model. A) The variable and constant RBC core model effective HRF shown is deconvolved from 12 minutes of white noise NO production when m = 4 Δdiameter,%ΔGCactivation,%. B) Convolution of the NO production rate with the kernel has a high predictive value for estimating the arteriole response for a different 12 minutes of data. R2 values shown are from m = 1,2,3,4, and 5. C) Example 300 seconds of data showing the difference between the response in the model and an approximation using the kernels shown in A. Example shown for m = 4. (TIF) [file pcbi.1008069.s008.tif]

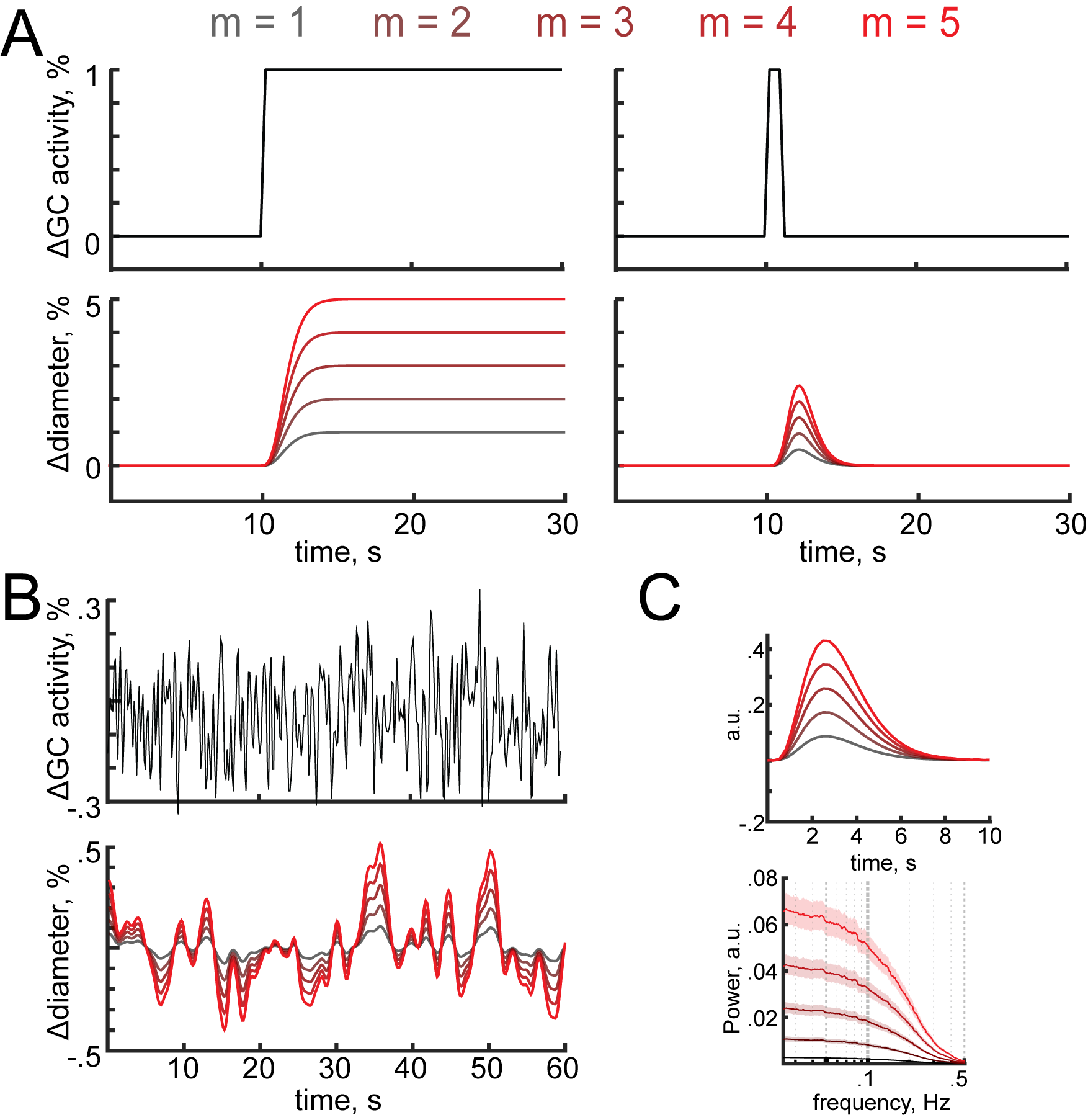

Supplement: S9 Fig — This model is solely a convolution of the kernel, h(t), with an imposed ΔGC activity and does not contain any COMSOL components that account for the diffusion of NO or geomsetry changes from arteriole dilation. A) A 1% increase in ΔGC activity (black) was imposed for 20 seconds or 1 second and convolved with the kernel, h(t), as shown in Eq 10 for varying degrees of arteriole sensitivity (m = 1,2,3,4 & 5). Although a larger “m” increased the magnitude of the expected vascular response (red), convolution of GC activity with the kernel was not able to generate the post-stimulus undershoot. B) Convolution of the kernel, h(t), with a white noise ΔGC activity (black) produced a predicted hemodynamic response (red). Only 60 seconds of the 50-minute simulation is shown. C) The HRF and power spectrum of the vascular response in B do not produce a post-stimulus undershoot or vasomotion-like oscillations, indicating that they are not emergent phenomena due to the chosen shape of h(t) or value of “m”. (TIF) [file pcbi.1008069.s009.tif]

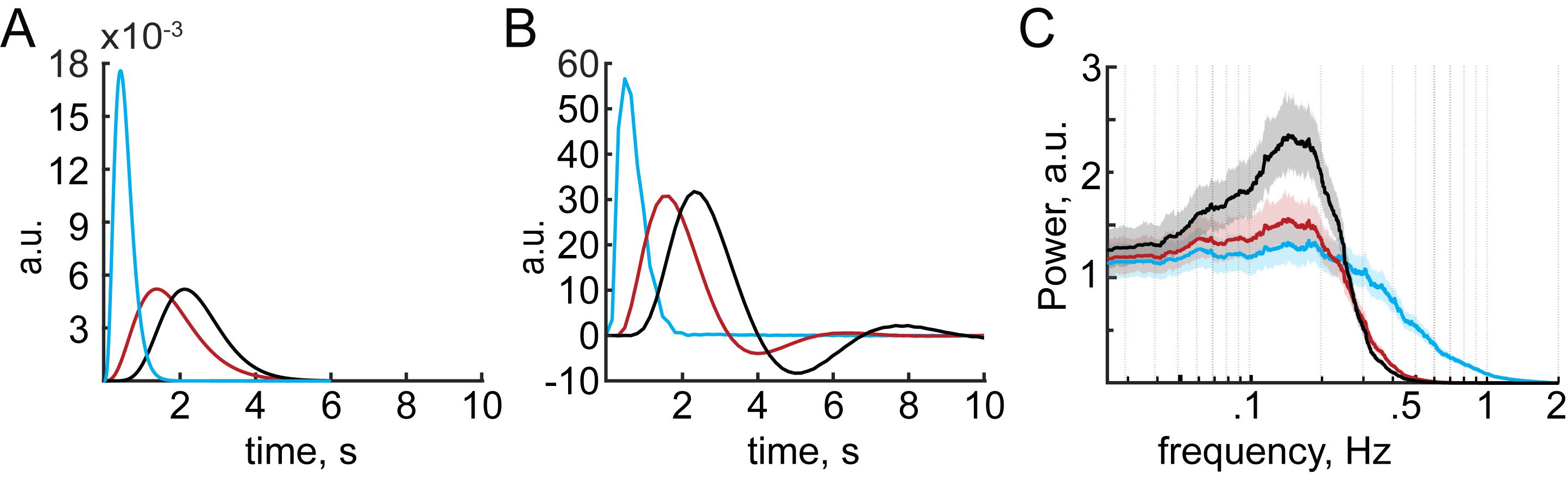

Supplement: S10 Fig — All simulations in this figure used a dynamic model. An arteriole sensitivity of m = 4 Δdiameter,%ΔGCactivation,% was used for these simulations. A) Plots of the HRFs used in these simulations. The HRFs used have faster (peak at 0.5 seconds, cyan) or slower (2.1 seconds, black) dynamics than the kernel used in all other simulations (1.5 seconds, red) Note that the integrated areas of all the HRFs are the same. B) Effective HRF evaluated from deconvolving the response to white noise NO stimulation. C) Power spectrum of the responses of each of the models to white noise. Slower hemodynamic responses to NO (black) amplified the post-stimulus undershoot and 0.1–0.3 Hz oscillations. The faster hemodynamic response (cyan) did not have an appreciable post-stimulus undershoot or resonant frequency near 0.1–0.3 Hz. Due to the rapid response to NO, the hemodynamics from the fast kernel contained power in the higher frequencies (C, cyan). (TIF) [file pcbi.1008069.s010.tif]

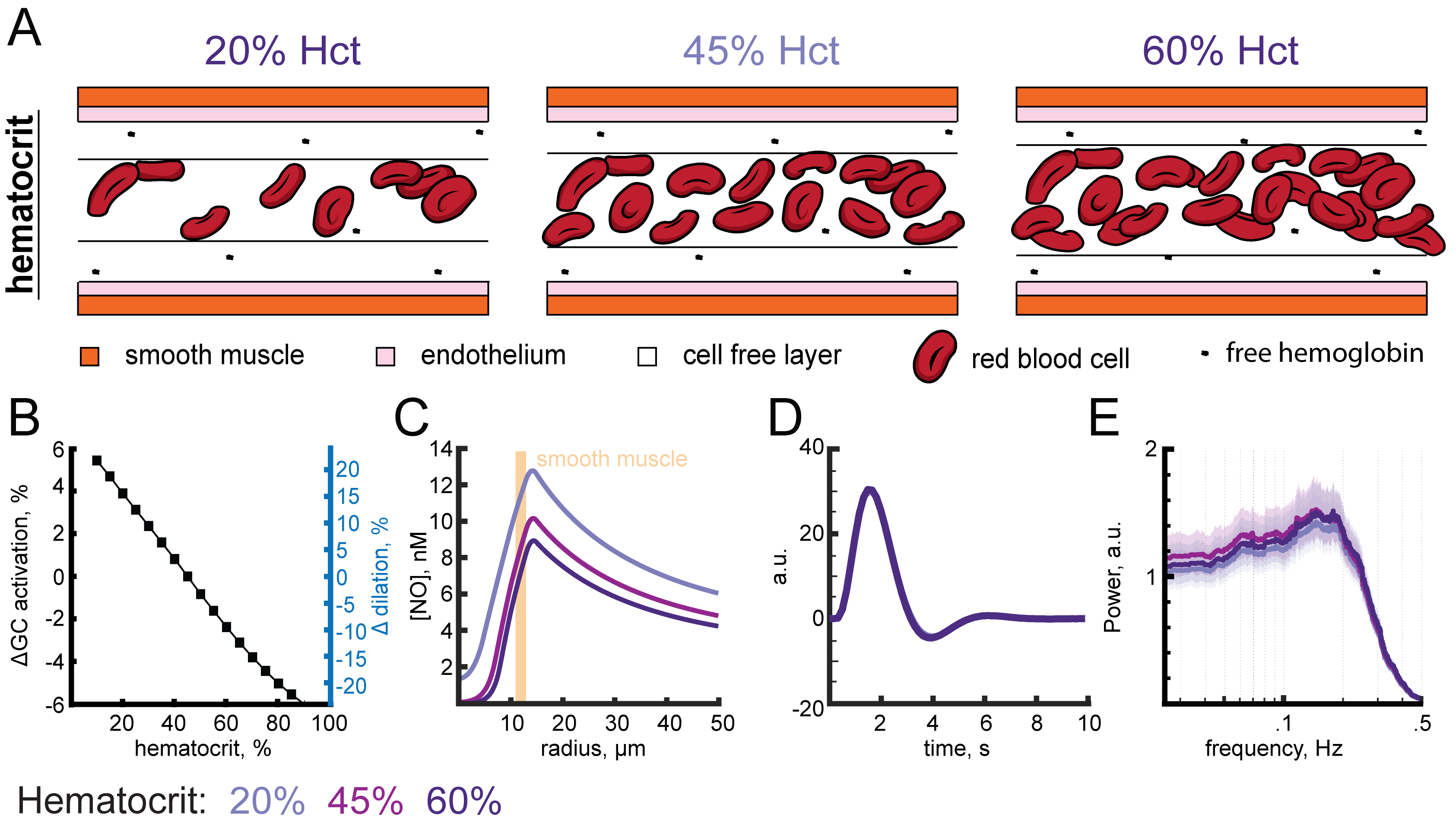

Supplement: S11 Fig — All simulations in this figure used a dynamic model. In this figure, the hematocrit was varied. A) Increasing hematocrit increases the degradation of NO in the RBC core and reduces the size of the cell free layer. B) Baseline GC activation and arteriole diameter are predicted to decrease with increasing hematocrit and increase with decreasing hematocrit. C) Perivascular NO increases with low hematocrit and decreases with elevated hematocrit. The location of the smooth muscle is indicated in orange. D) Changing hematocrit does not alter the hemodynamic response function (note near complete overlap of effective HRFs) or the frequency response of the vessels (E). (TIF) [file pcbi.1008069.s011.tif]

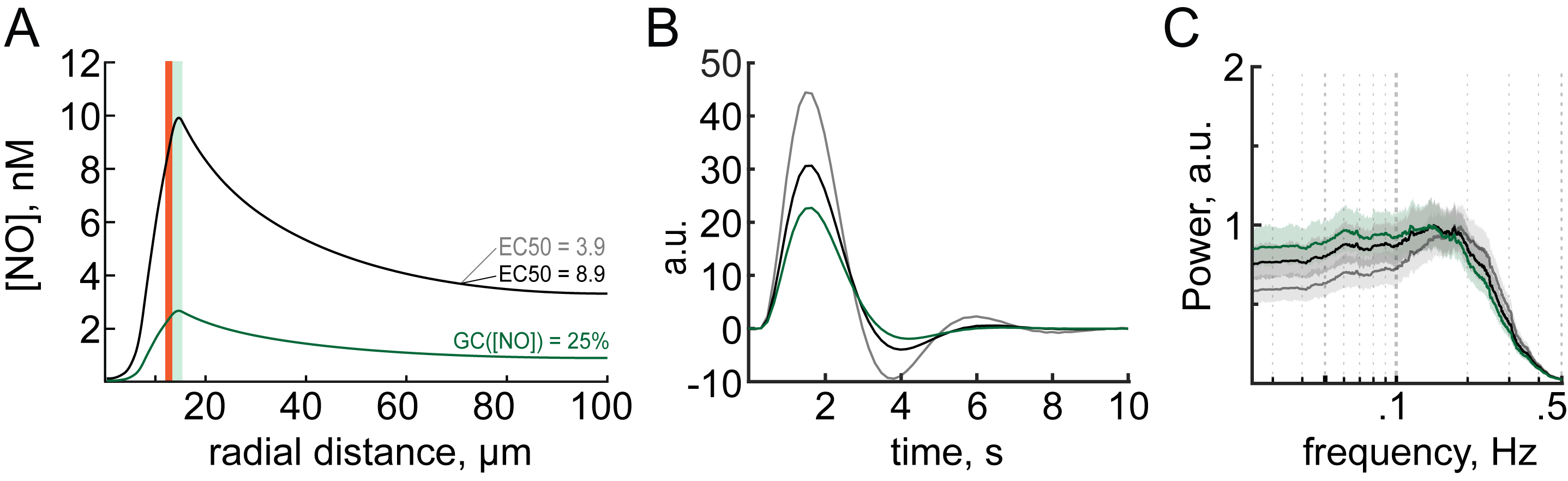

Supplement: S12 Fig — All simulations in this figure used a dynamic model. An arteriole sensitivity of m = 4 Δdiameter,%ΔGCactivation,% was used. A) The perivascular NO concentration was evaluated for a baseline NO concentration eliciting 25% activation of the smooth muscle (green) or 50% GC activity with an EC50 of 3.9 nM and Hill coefficient of 2.1 [149] (gray). The simulation with an EC50 of 8.9 nM (Hill coefficient, 0.8) and baseline GC activity of 50% is shown in black for reference. Black and gray perivascular NO profiles overlap because the NO production rate was set such that there was 50% GC activity in the smooth muscle for both cases. B) Effective HRFs obtained by deconvolution of the hemodynamic response to white noise NO production. C) Power spectrum of each of the three conditions in response to white noise NO production. Decreasing baseline GC activity reduced the magnitude of the post-stimulus undershoot and bandpass-like properties of the system (green). Decreasing the EC50 of the relationship between NO and GC activity and increasing the Hill coefficient to 2.1 amplified the post-stimulus undershoot and bandpass-like properties (gray). (TIF) [file pcbi.1008069.s012.tif]

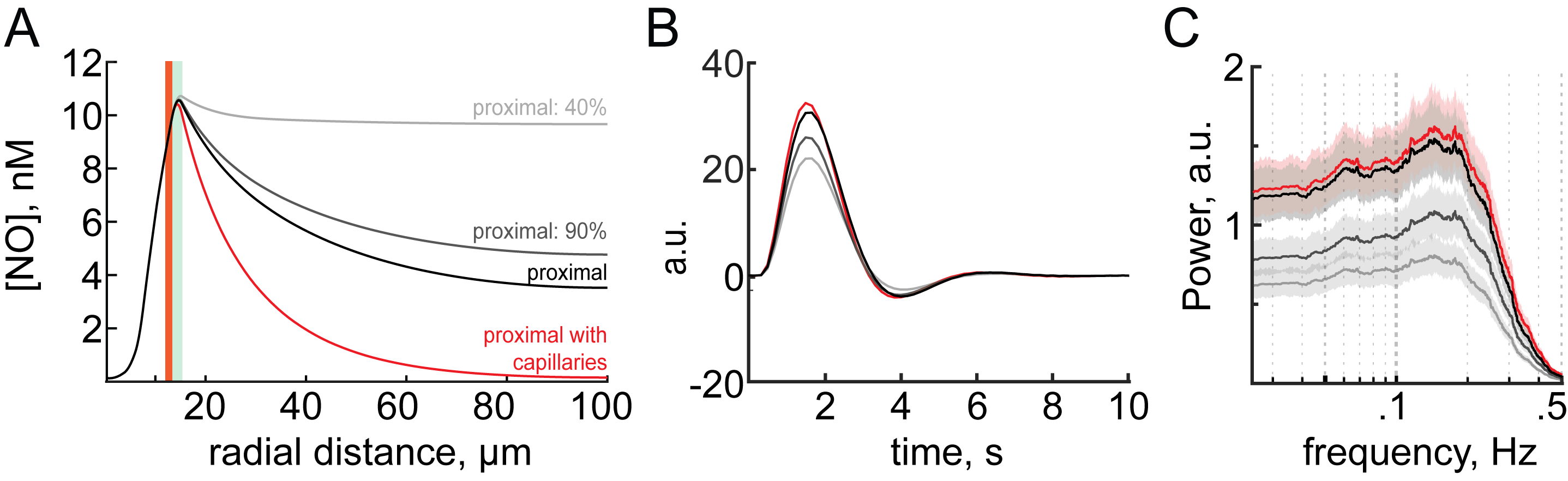

Supplement: S13 Fig — All simulations from this figure used a dynamic model. An arteriole sensitivity of m = 4 Δdiameter,%ΔGCactivation,% was used. A) The perivascular NO concentration was evaluated with the presence of capillaries (red) or with increasing amounts of background NO production (gray and light gray). The rates of NO production by the parenchyma distal to the proximal 2 μm of the arteriole was adjusted to compose either 0% (black), 10% (gray) or 40% (light gray) of the total NO produced. As a larger fraction of NO is produced distally, perivascular NO concentrations increase (gray and light gray) while the presence of capillaries depletes perivascular NO (red). B & C) B and C were evaluated by deconvolution of the hemodynamic response with a white noise NO production rate or by reporting the frequency spectrum of the hemodynamics respectively. The presence of capillaries (red) or an increased rate of background NO production (gray and light gray) had minimal effects on the undershoot present in the effective HRF or bandpass-like properties of the system from the proximal simulation (black). (TIF) [file pcbi.1008069.s013.tif]
